# The second generation of The Avon Longitudinal Study of Parents and Children (ALSPAC-G2): a cohort profile

**DOI:** 10.12688/wellcomeopenres.15087.2

**Published:** 2019-12-16

**Authors:** Deborah A. Lawlor, Melanie Lewcock, Louise Rena-Jones, Claire Rollings, Vikki Yip, Daniel Smith, Rebecca M. Pearson, Laura Johnson, Louise A. C. Millard, Nashita Patel, Andy Skinner, Kate Tilling, ALSPAC Executive

**Affiliations:** 1 MRC Integrative Epidemiology Unit at the University of Bristol, University of Bristol, Bristol, BS8 2BN, UK; 2 Population Health Science, Bristol Medical School, University of Bristol, Bristol, UK; 3 NIHR Bristol Biomedical Research Centre, Bristol, UK; 4 ALSPAC, University of Bristol, Bristol, UK; 5 Centre for Academic Mental Health, University of Bristol, Bristol, UK; 6 Centre for Exercise, Nutrition and Health Science, School for Policy Studies, University of Bristol, Bristol, UK; 7 Intelligent Systems Laboratory, University of Bristol, Bristol, UK; 8 Department of Women and Children’s Health, School of Life Course Sciences, Kings College London, London, UK

**Keywords:** ALSPAC, Birth Cohort, Cross-generation, Data Sharing

## Abstract

**Background: **The Avon Longitudinal Study of Parents and Children-Generation 2 (ALSPAC-G2) was set up to provide a unique multi-generational cohort. It builds on the existing ALSPAC resource, which recruited 14,541 pregnancies to women resident in the South West of England who were expected to deliver between 01/04/1991 and 31/12/1992. Those women and their partners (Generation 0; ALSPAC-G0) and their offspring (ALSPAC-G1) have been followed for the last 27 years. This profile describes recruitment and data collection on the next generation (ALSPAC-G2)—the grandchildren of ALSPAC-G0 and children of ALSPAC-G1.

**Recruitment:** Recruitment began on the 6 ^th^ of June 2012 and we present details of recruitment and participants up to 30 ^th^ June 2018 (~6 years). We knew at the start of recruitment that some ALSPAC-G1 participants had already become parents and ALSPAC-G2 is an open cohort; we recruit at any age. We hope to continue recruiting until all ALSPAC-G1 participants have completed their families. Up to 30 ^th^ June 2018 we recruited 810 ALSPAC-G2 participants from 548 families. Of these 810, 389 (48%) were recruited during their mother’s pregnancy, 287 (35%) before age 3 years, 104 (13%) between 3-6 years and 30 (4%) after 6 years. Over 70% of those invited to early pregnancy, late pregnancy, second week of life, 6-, 12- and 24-month assessments (whether for their recruitment, or a follow-up, visit) have attended, with attendance being over 60% for subsequent visits up to 7 years (too few are eligible for the 9- and 11-year assessments to analyse).

**Data collection: **We collect a wide-range of socioeconomic, lifestyle, clinical, anthropometric and biological data on all family members repeatedly. Biological samples include blood (including cord-blood), urine, meconium and faeces, and placental tissue. In subgroups detailed data collection, such as continuous glucose monitoring and videos of parent-child interactions, are being collected.

## Introduction

### Why was the cohort set up?

The Avon Longitudinal Study of Parents and Children-2 ^nd^ Generation study (ALSPAC-G2) was set up to provide a unique multi-generational family study and to be a resource for international researchers to explore the environmental, socioeconomic, lifestyle, physiological, metabolic, genomic and epigenomic contributions to health and development across the lifecourse and across generations. It builds on the existing ALSPAC resource which originally recruited 14,541 pregnancies to women who were resident in the former county of Avon (centred around the city of Bristol in the South West of England) and who were expected to deliver between 01/04/1991 and 31/12/1992. Those women and their partners (ALSPAC-G0), together with their index children (ALSPAC-G1), who are now in their late-20s, have been followed since pregnancy or birth, with full details provided in previous cohort profiles ^1, 2^. The ALSPAC resource, including ALSPAC-G2, receives core funding from the University of Bristol, Wellcome and UK Medical Research Council, with additional support from a very wide range of national and international funders (a comprehensive list of grant funding is available on the ALSPAC website: http://www.bristol.ac.uk/alspac/external/documents/grant-acknowledgements.pdf). Since its inception in the early 1990s the study has been known by two names. ALSPAC, including ALSPAC-G2, is used in all academic publications, presentations and with research funders. To participants and in the media the original study (specifically ALSPAC-G1) is known as Children of the 90s (Co90s) and ALSPAC-G2 is known as Children of the Children of the 90s (CoCo90s; sometimes abbreviated verbally to CoCos).

It was envisaged that initially ALSPAC-G2 would contribute unique research in the following broad areas:

1. Understanding how socioeconomic, lifestyle, patho-physiological, metabolic, genomic and epigenomic factors combine to influence the associations of health and wellbeing across generations. With the addition of ALSPAC-G2, ALSPAC is unique in being the only human study able to do this with relevant detailed data across three generations.
2. Determining the impact of pre-conceptual health and wellbeing of mothers and fathers on fertility and growth, development and health of their offspring. The importance of pre-conceptual cohort studies is increasingly recognised but they are difficult to establish, with many existing pre-conceptual cohorts recruited from fertility clinics rather than the general population as here ^3^. For ALSPAC-G2 children, we have detailed repeatedly assessed preconceptual data for at least one of the parents (the original ALSPAC-G1 participant who has become a parent). For the second parent who was not originally in ALSPAC but is recruited to ALSPAC-G1 as part of extending the resource to ALSPAC-G2 we will have pre-conceptual data from record linkage and for subsequent pregnancies/children (i.e. those recruited after the first child who is recruited to ALSPAC-G2) we will also have preconceptual data from our research clinic. No existing birth cohort has such extensive parental data, including on fathers, with many having little or no data on large proportions of fathers ^4^.
3. Understanding the impact of major changes that have occurred over the last 20–25 years and how these have influenced differences between ALSPAC-G0 and ALSPAC-G1 in relation to whether, and when, to start a family, parenting patterns, and ‘work-family/outside of work’ balance and of these on differences in health and development of ALSPAC-G1 and ALSPAC-G2. These changes have been unprecedented and include changes in environmental (e.g. climate change, air-pollution), societal (e.g. different methods of communication, changing patterns of between and within country migration), political (e.g. distrust of experts, departures from traditional political parties, changes in gender politics, retirement age and types of employment) technological (e.g. widespread use of information technology and the emergence of artificial intelligence) and lifestyle (e.g. sedentary behaviour, reduced smoking and alcohol consumption, increased vaping) factors. For example, ALSPAC-G0 participants mostly finished their education and started their careers before the internet was invented. In its current recognisable form, widespread availability of the internet began in 1990 just before ALSPAC-G1 were born but its reach into primary schools will have been limited for this generation. In contrast, ALSPAC-G2 are likely to spend their whole lives extensively using the internet for their education, work and social life.
4. Providing an opportunity to explore factors associated with, and outcomes of, starting a family at a relatively young or relatively older age in comparison to the most common age range for the generation that ALSPAC-G1 reflect. Understanding how differences in parental age at starting a family and the factors associated with that influence health and wellbeing in the generation that ALSAPC-G2 reflect. This will be facilitated by collecting data on all children of a well characterised cohort born at the same time (1991–92) in the same geographical area (South West of England) and hence removing confounding by period and location of birth.
5. Exploiting the very detailed genetic and phenotypic data collected on parents and siblings to improve causal inference in research, by triangulating results using different methodological approaches, such as Mendelian randomization, parental negative control studies and within sibship analyses ^5^.
6. Providing a platform for pilot and feasibility studies of novel technologies for data collection, such as the use of non-intrusive wearables (e.g. biosensors and smart watches) for continually monitoring physiology and behaviours.

As ALSPAC-G2 participants will take another ~20 years to recruit, the extent to which each of these research areas can be addressed will vary over time. It will also depend on funding availability and continued participant engagement. Considering the ‘short-term’ to refer to research that is, or could be, done currently, medium-term what will be completed in the next 5 to to 10 years and longer-term what will be mostly done in the next 10–20+ years, ALSPAC-G2 has already contributed to the first area of research listed above (see section “What has ALSPAC-G2 found? Key findings and publications”) and, with continued funding secured for a further 5 years, will continue to contribute to this area in the medium-term. Similarly, ALSPAC-G2 will contribute to research areas 2, 3 and 6 in the short- and medium-term. Area 4 is not possible until the ALSPAC-G1 participants who choose to become parents have completed their families and is therefore a long-term ambition. Some of the causal analyses listed in area 5 require large sample sizes for precise estimation. For continuously measured outcomes ALSPAC-G2 will be able to contribute to within sibship and parental negative control studies within the next 5 years (i.e. in the recently awarded funding period) ALSPAC-G2 will be able to contribute to genome-wide and Mendelian randomization analyses, including family based Mendelian randomization through participation in large collaborations, such as the Early Growth Genetics (EGG) consortia, which already includes cohorts with fewer participants than currently in ALSPAC-G2 ^6, 7^. If we continue to be funded beyond 2024 (i.e. the end of the current core funded period), and engage participants, the study will contribute translational research to all areas with unique data on families where data on at least one parent will be known from when they were in utero to when they completed their family. We describe varying sources of bias over this time of recruitment in the section on “What are the main strengths and limitations”.

## Who is in the cohort?

**Figure 1.**
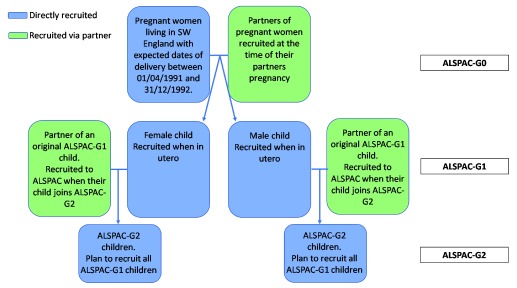
Summarising timing and nature of recruitment to different ALSPAC generations.

We began recruitment to ALSPAC-G2 on the 6 ^th^ June 2012. Our aim is to recruit all children of ALSPAC-G1 participants into ALSPAC-G2. As well as recruiting the next generation of children, we also recruit the (non-ALSPAC) partners of their ALSPAC-G1 parent and collect data from them ( Figure 1). Of the 810 ALSPAC-G2 pregnancies/children recruited to date both parents of 74 (9%) were original ALSPAC-G1 children. Thus, for each ALSPAC-G2 pregnancy/child we have very detailed repeatedly assessed information on at least one of their parents from when that parent was *in utero* to the time of them becoming pregnant/a parent (with this being the case for both parents for 9% of ALSPAC-G2). We also have newly collected information from questionnaires, clinic assessments, record linkage and blood samples (on which genome-wide, epigenetic and metabolomic data are being assessed), from both parents, including any parent who was not an original ALSPAC-G1 participant, but is recruited to be part of ALSPAC-G1 as we recruit their ALSPAC-G2 child.

We recruit ALSPAC-G2 participants through multiple means: via questionnaires to all ALSPAC-G0 and -G1 participants, in which we ask about becoming pregnant or a parent (ALSPAC-G1) or a grandparent (ALSPAC-G0); asking similar questions when any ALSPAC-G0 or -G1 participant attends a research clinic visit; and through posters in primary care clinics and maternity units in the South West of England. We also use our regular mailed and emailed newsletters and conventional (e.g. local radio and newspapers) and social (e.g. Facebook, Instagram and Twitter) media to remind ALSPAC-G0 and -G1 participants about ALSPAC-G2. When we hear about an ALSPAC-G1 participant becoming pregnant or a parent from one of their parents (ALSPAC-G0) we ask that their parent mention ALSPAC-G2 to them and ask them to contact us if they are interested in hearing more about the study . Similarly, when ALSPAC-G1 participants contact us because of hearing about the study via our newsletter or other media, we ask if they would like an information pack. Parents of ALSPAC-G2 participants can be resident anywhere, though the vast majority remain in the UK; we offer travel costs to clinic visits in Bristol. Biological samples at birth (cord-blood and placental tissue) are currently collected on participants delivering at one of nine maternity units in and around the South West of England.

***Ethics***. Ethical approval for ALSPAC was obtained from the ALSPAC Ethics and Law Committee and the UK National Health Service Research Ethics Committee (full details are available at: http://www.bristol.ac.uk/alspac/researchers/data-access/ethics/lrec-approvals/#d.en.164120). Participants (the main care-giver for children) provided written informed consent for data collection and its use in research.

**Figure 2.**
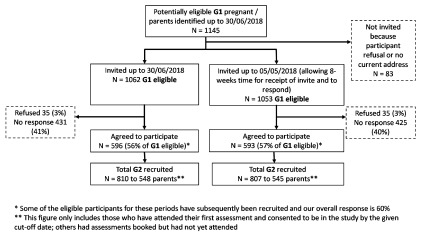
ALSPAC-G2 recruitment from first recruitment (06/06/2012) to 30/06/2018.

***Response and characteristics of eligible participants who are and are not recruited***. For all analyses in this profile we have restricted the sample to children of any ALSPAC-G1 pregnant/parent participant who agreed to be sent an invitation pack and for whom we had a correct address ( Figure 2) up to and including 30/06/2018. This provides just over 6 years of data and means we can include all data that has had appropriate quality control checks and linkage to the existing ALSPAC-G0 and -G1 data.

Over this period, we have identified 1145 ALSPAC-G1 participants who were pregnant, had a partner who was pregnant or had become a parent, and we sent invites to 1062 (93%) of these, with 596 (56% of those invited) agreeing to participate. By 30 ^th^ June 2018 we had assessed and obtained parental consent for 810 ALSPAC-G2 children from 548 families ( Figure 2). Of these 810 ALSPAC-G2 participants 389 (48%) were recruited in either early or late pregnancy, with the proportion recruited in pregnancy increasing over time from 20% to 63% between the first year (6 ^th^ June 2012 to 30 ^th^ June 2013) and the most recent year (1 ^st^ July 2017 to 30 ^th^ June 2018) ( Table 1). As might be expected, none of the ALSPAC-G2 participants were recruited at the 7–15-days assessment; all participants seen at 7–15-days were recruited in pregnancy. Similarly, none of the ALSPAC-G2 participants were recruited at the 11-year assessment; those seen at that age were all recruited at earlier ages ( Table 1).

**Table 1.** Number of participants recruited at different assessment ages.

Participant recruitment stage	Number of ALSPAC-G2 recruited at each age by year of recruitment (% recruited at each age by each year)						
	Year 1 6 ^th^ June 2012–30 ^th^ June 2013, n (%)	Year 2 1 ^st^ July 2013–30 ^th^ June 2014, n (%)	Year 3 1 ^st^ July 2014–30 ^th^ June 2015, n (%)	Year 4 1 ^st^ July 2015–30 ^th^ June 2016, n (%)	Year 5 1 ^st^ July 2016–30 ^th^ June 2017, n (%)	Year 6 1 ^st^ July 2017–30 ^th^ June 2018, n (%)	Total recruited at each age 6 ^th^ June 2012–30 ^th^ June 2018
Early pregnancy ^a^	8 (6)	30 (25)	21 (23)	51 (33)	53 (32)	68 (44)	231
Late pregnancy ^a^	18 (14)	23 (19)	27 (30)	28 (18)	32 (20)	30 (19)	158
*Total pregnancy* ^a^	*24 (20)*	*53 (44)*	*48 (53)*	*79 (51)*	*85 (52)*	*98 (63)*	
7–15 days	0 (0)	0 (0)	0 (0)	0 (0)	0 (0)	0 (0)	0
6 months	30 (24)	19 (16)	17 (19)	35 (23)	18 (11)	18 (12)	137
12 months	26 (21)	18 (15)	5 (6)	7 (5)	15 (9)	8 (5)	79
24 months	18 (14)	13 (11)	10 (11)	13 (9)	7 (4)	10 (6)	71
36 months	14 (11)	9 (8)	3 (3)	5 (3)	13 (8)	8 (5)	52
48 months	6 (5)	3 (3)	4 (4)	7 (5)	13 (8)	0 (0)	33
60 months	4 (3)	4 (3)	2 (2)	2 (1)	4 (2)	3 (2)	19
6 years	3 (2)	0 (0)	0 (0)	2 (1)	2 (1)	5 (3)	12
7 years	0 (0)	1 (1)	1 (1)	3 (2)	6 (4)	2 (1)	13
9 years	0 (0)	0 (0)	0 (0)	0 (0)	1 (1)	4 (3)	5
11 years	0 (0)	0 (0)	0 (0)	0 (0)	0 (0)	0 (0)	0
Total recruited in each year	127 (100)	120 (100)	90 (100)	153 (100)	164 (100)	156 (100)	810

^a^ For the period covered in this paper early pregnancy was defined as up to 18-weeks of complete gestation and later pregnancy as 28- or more weeks. However, we have been flexible with these definitions to maximise recruitment and minimise participant burden and found that we see women across all gestations (including between 18- and 28-weeks gestation), thus those included in the early pregnancy category will include women up to 23-weeks and those as late 24- or more weeks. We are currently changing our protocols to having just one assessment during pregnancy at whatever gestational age best suits the woman and her family (see also text in section about how often participants are followed-up). For this reason, we have also provided the total numbers and percentage recruited at any time in pregnancy (in italics).

## Characteristics of those recruited

In analyses using observed data and not taking account of missing data, eligible ALSPAC-G1 parents/pregnant women who were recruited were slightly younger, had higher BMI and were more likely to have attended the two most recent clinic assessments (17–18 or 23–24 years) than those who declined or did not respond, but were similar in terms of sex, educational attainment, smoking and whether they lived in the Bristol area at the time of recruitment ( Table 2). However, there was more missing data for these characteristics among those who were not recruited. We therefore used multiple imputation to explore the extent to which missing data may have biased these results. Missing values were imputed using chained equations based on ALSPAC-G1 adolescent BMI, and characteristics of their mothers (i.e. ALSPAC-G0; educational attainment, home ownership, occupational social class, parity, smoking during pregnancy, pre-pregnancy body mass index, age at birth of their ALSPAC-G1 child) in addition to all variables included in the association analyses presented in Table 2 ^8^. For each variable with missing data, 100 imputed variables were created. Linear regression was used to impute continuous variables, logistic for binary, and multinomial logistic for multi-category variables ^8^. Differences (differences in means or odds ratios) between characteristics were estimated in each of the 100 imputed datasets and then pooled using Rubin’s rules ^8^. The results based on observed and imputed data were similar ( Table 2).

**Table 2.** Comparison of those recruited and those who have declined or not responded. All results are all from questionnaires, research clinic assessments or record linkage data on a ALSPAC-G1 participant who has become pregnant, or for male ALSPAC-G1 has a partner who has become pregnant or who had become a parent and were eligible to be recruited to ALSPAC-G2 up to 30 ^th^ June 2018.

	Recruited ^a^ (N = 580)		Declined or did not respond ^b^ (N = 447)			
*Continuous outcomes*	N (%) with data	Mean (SD)	N (%) with data	Mean (SD)	Difference in mean (95%CI) ^c^ Observed (complete) data	Difference in mean (95%CI) ^c^ Multiple imputed data
Age (Years) ^d^	580 (100)	22.5 (1.9)	446 (100)	22.8 (1.9)	0.37 (0.13, 0.60)	0.37 (0.13, 0.60)
BMI (Kg/m ^2^) ^e^	467 (81)	26.3 (6.1)	215 (48)	25.4 (5.7)	-0.87 (-1.8, 0.1)	-0.89 (-1.79, -0.01)
	Recruited ^a^ (N = 580)		Declined or did not respond ^b^ (N = 447)			
*Binary outcomes*	N (%) with data	Number with the outcome (%)	N (%) with data	Number with the outcome (SD)	Odds ratio (95%CI) ^c^ Observed (complete) data	Odds ratio (95%CI) ^c^ Multiple imputed data
Female	580 (100)	444 (77)	447 (100)	329 (74)	0.85 (0.64, 1.14)	No missing data
Educational attainment to A-level or higher ^f^	401 (69)	218 (54)	222 (50)	115 (52)	0.9 (0.65, 1.25)	0.86 (0.62, 1.19)
Ever smoked ^g^	509 (88)	336 (66)	252 (56)	170 (67)	1.07 (0.77, 1.47)	1.08 (0.78, 1.48)
Attended 17/18-year or 24/25-year follow-up	568 (98)	472 (83)	441 (99)	221 (50)	0.2 (0.15, 0.27)	0.2 (0.15, 0.27)
Living in Bristol at time of (potential) enrolment	579 (100)	476 (82)	446 (100)	368 (83)	1.02 (0.74, 1.41)	1.02 (0.74, 1.41)

^a^ ALSPAC-G1 participants who were invited and have attended at least one assessment; ^b^ ALSPAC-G1 participants who were invited and have declined or not responded; ^c ^Differences in means for age and BMI, odds ratios for educational attainment, smoking and attendance at recent clinics, in all analyses those recruited are the reference category; ^d^ At time of invitation; ^e^ At either of the two most recent assessments 24/25 years for those with data from that follow-up, otherwise 17/18 years; ^f ^A-levels: Advanced-levels, secondary school exams required for University entrance and some other further education/apprentice schemes taken at age 18. Data obtained from record linkage; ^g^ Data from any previous questionnaire (data on smoking have been collected repeatedly since age 17 years). ^h^ based on having a Bristol (BS) postcode at the time when the ALSPAC-G2 enrolment pack was sent.

## How often have they been followed-up?

**Figure 3.**
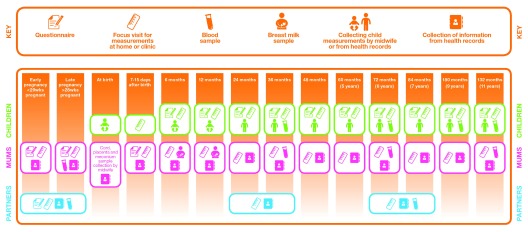
Summary of data collected on parents and children in ALSPAC-G2. Figure 3 summarises data collection times and type for the period covered by this profile i.e. from initiation of ALSPAC-G2 in June 2012 to the end of June 2018. With the new renewal for a further five years, we are now undertaking just one assessment during pregnancy and this will be at a time that best suits the pregnant woman.

From the start of the study, we established ALSPAC-G2 as an open cohort: open both in terms of when someone might enter the study (i.e. mothers’ pregnancy, infancy, childhood or later) and the length of time we aim to keep recruitment open for ALSPAC-G2 (and subsequent generations). We currently have protocols for data collection (on both parents (ALSPAC-G1) and their offspring (ALSPAC-G2)) for early pregnancy (up to 20 completed weeks of gestation), late pregnancy (≥28 weeks gestation), first 7–15 days, 6-months, 12 months, annually up to 7 years and then at 9 and 11 years ( Figure 3). Our current plans are to extend this with protocols for data collection every 2 years up to the age of 21 over the next 5-years. We also plan to have just one ‘pregnancy’ assessment at any time during pregnancy (see below).

Participants are invited to all subsequent assessments after the first one that they attend. Both at recruitment and follow-up we are flexible about which assessment age they ‘slot’ into in order to maximise response and minimise participant burden, particularly when ALSPAC-G1 parents have more than one ALSPAC-G2 child. For example, an ALSPAC-G1 woman who was recruited when 21 weeks pregnant with a second child and who already had a 31-month-old child in ALSPAC-G2, would have the pregnancy assessment for their 2 ^nd^ pregnancy at the same time as the next assessment due for the first child. That is, we would do the 36-month assessment for the first child a little earlier than had their mother not been pregnant with a second child to minimise participant burden. The family would subsequently be eligible for annual child assessments of the older child and birth, 6-months and then annual child assessments of the younger child. We would plan all subsequent visits so that the family would only need to attend once for each of these subsequent assessments of both children. At all assessments we would complete relevant parental and children assessments and within the study database record this. For example, at the assessments where the mother is pregnant with a second child and also due to have a ~36-month postnatal assessment in relation to their first child, results would be linked to both children and we would do all 36-month measurements (that were feasible in a pregnant woman), as well as all pregnancy measurements. For the 36-month postnatal measurements we would indicate that the woman was pregnant with another child.

We have found that we see many ALSPAC-G1 pregnant women between the thresholds that we initially used to defined early and late pregnancy (i.e. between 20 and 28 weeks of gestation). This is because once women contact us to join the study they are keen to make an appointment as soon as possible at a date that suits them, and also because of combining assessments when parents have more than one ALSPAC-G2 child (see example description above). We therefore plan to change to having a protocol for recruitment at any gestational age in pregnancy and to see women just once during pregnancy.

**Figure 4.**
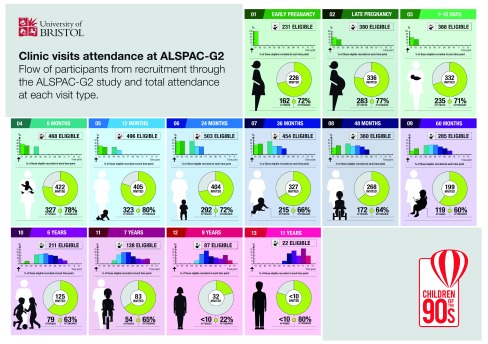
Summary of eligible and invited participants at each assessment time. Each section in this figure represents one of the ALSPAC-G2 age periods (from early pregnancy to 11 years) at which we currently collect data. In each section, for each assessment we report the number eligible and the composition of that eligible number based on when they were first invited and recruited to the study (bar graph at the top of each assessment period). We also show the total number invited to that assessment and the number and percentage of those invited who attended (central figure). All numbers in this figure refer to invites and recruitment between June 2012 and June 2018.

In an open cohort like ALSPAC-G2 in which participants may be initially recruited at different ages from when they are *in utero* early in their mother’s pregnancy to late childhood, follow-up rates can be difficult to describe. This is because at each of the ALSPAC-G2 assessment ages eligible participants include any fetus/infant/child who is being recruited and seen for the first time at that age as well as those who were recruited at any of the earlier assessment times who have reached the age of the assessment under consideration. We summarise this in Figure 4. Taking 12 months as an example, we can see that over the period covered by this cohort profile, 496 ALSPAC-G2 participants were eligible for a 12-month assessment and of those 31% had been recruited in early pregnancy, 26% in late pregnancy, 26% at 6-months and 17% were being recruited for the first time at 12 months. In total 405 had been invited by June 2018 and of those 323 (80%) were assessed. For the first six assessment periods (early pregnancy to 24 months), 71–80% of those invited have attended and attendance remains at 60% or higher for all assessments up to 7-years. For the two oldest ages at which we currently assess ALSPAC-G2 participants, 9 and 11 years, the proportions are 22% and 80%, respectively. The low response at 9 years was because of delays in developing protocols and gaining ethical approval for the later assessment periods, which meant that some had become closer to 11 years and were seen for the first time at that assessment. That said, for both the 9- and 11-year assessments numbers of invited participants are low, and the percent assessed less reliable than at younger ages.

## What has been measured?

We use REDCap software for direct data entry at the point of collection. Figure 3 summarises the core data that was collected between June 2012 and June 2018 on parents (ALSPAC-G1) and their offspring (ALSPAC-G2). More detailed lists of variable types are provided in the appendices. These list the information collected via questionnaires, clinical assessments, extraction of data from clinical records and record linkage, and also list the stored biosamples we have for mothers, fathers and the ALSPAC-G2 children (see Extended Data, Summary of data collection) ^9^.

We collect a range of environmental, socioeconomic, lifestyle, clinical and biological data on all family members repeatedly ( Figure 3). Biological samples collected routinely include blood (including cord-blood), urine, meconium and faeces, and placental tissue. Samples are collected with broad generic consent to enable a wide range of future use, including genetic analyses. Up to June 2018, we also invited all relevant mothers (those recruited during pregnancy, 6- and/or 12-months postnatal, and who breastfed) to provide repeat breast milk samples as part of a pilot study to assess response to this request. In total 457 mothers of 593 children (taking account of siblings and twins) were eligible and of these, 137 mothers (of 168 children) gave consent to participate in this pilot and 105 (of 121 children) have provided at least one breast milk sample. Of the 105 mothers who provided breast milk, 90 gave sample(s) for 1 infant, 14 gave for 2 infants, and 1 for 3 infants. For any given infant the range of samples that have been obtained is 1 to 4 repeats. To date, no analyses have been performed on these samples (we would be keen to speak to any collaborators who would like to use these samples). We have also collected, or are collecting, intensive repeat or continuously measured data, using novel methods on sub-groups, including continuously measured glucose on mothers during pregnancy ^10^, videos of parent-child interactions from head-worn and home-based cameras ^11^, and dietary intake from smart-phone photographs of meals ^12^.

## Participant engagement and involvement

We have had active participant groups in ALSPAC since it began. These groups suggest areas of data collection and provide advice on planned data collection, including methods for collecting these data. In addition, we receive direct advice and suggestions as we collect data (questionnaires or clinic assessments). For ALSPAC-G2, this has included help with collecting and appropriately labelling data for same-sex couples and suggesting that we collect data on baby-led weaning and helping us to develop appropriate questions about this. From age 9 years we involve the ALSPAC-G2 children directly via a participant information sheet that they (and their parents) helped us to design (see Extended Data, Child PIS) ^9^.

## What has ALSPAC-G2 found? Key findings and publications

***Cross-generational comparisons of antenatal and infancy characteristics***. In pregnant women aged 19–24 years, we have shown that the risk of antenatal depressive symptoms is 50% higher in young women who were pregnant between 2012 and 2016, compared with their mother’s generation who were pregnant between 1990 and 1992 (relative risk 1.51 [95% confidence interval: 1.11, 2.05]), with these findings remaining unchanged in numerous sensitivity analyses, including when restricting analyses to 66 mother-offspring pairs ^13^. When individual symptoms were examined, the contemporary generation reported notably higher levels of feeling overwhelmed, crying often and having difficulty sleeping. Offspring of mothers who experienced high levels of antenatal depressive symptoms were over three times more likely to also experience high levels of symptoms ^13^.

We have undertaken additional preliminary cross-generational analyses of pregnancy and perinatal outcomes for this cohort-profile. In these analyses we only included ALSPAC-G0 women who were aged 19–26 at the birth of their ALSPAC-G1 child, as over 95% of the ALSPAC-G1 women were within this age range when pregnant with their ALSPAC-G2 child. In both generations we only included the first pregnancy recruited to the study. For these preliminary analyses we have only adjusted for maternal age (in years) and we used robust standard errors because of non-independence between the 197 mother-daughter (ALSPAC-G0-ALSPAC-G1) pairs across the two groups.

We imputed missing data in two ways; one in which both generations ALSPAC-G0/G1 and -G1/G2 were combined (treated as one cohort) and then imputation was carried out and one in which imputation was carried out separately in each generation and then imputed datasets were combined before the main analyses were undertaken. The former assumes there are no interactions in associations between the two generations, whereas the latter allows for this. In both approaches we used chained equations to predict missing data and for each variable with missing data 100 imputed variables were created (i.e. 100 imputed datasets were generated). Linear regression was used to impute continuous variables, logistic for binary, and multinomial logistic for multi-category variables ^8^.

The imputation model in the combined imputation analyses included all variables used in any of the cross-cohort association analyses shown in the final column of Table 3, with the units and categories for these variables being as shown in Table 3. In addition the following *a priori* selected predictors of missing data were included in the imputation models: whether ALSPAC-G0 or ALSPAC-G1 ever smoked (outside of pregnancy), the ALSPAC-G0’s and ALSPAC-G1’s mother’s educational attainment (i.e., for ALSPAC-G0/G1 pregnancies whether the ALSAPC-G0’s mother had any A-levels and for G1/G2 pregnancies whether the ALSPAC-G1’s mother had any A-levels), the ALSPAC-G0’s and ALSPAC-G1’s mother’s occupational social class (i.e., for ALSPAC-G0/G1 pregnancies this was the ALSPAC-G0’s mother’s social class, while for ALSPAC-G1/G2 pregnancies this was the ALSPAC-G1’s mother’s social class; social class was categorised as either “Professional/Managerial and technical", “Skilled non-manual", "Skilled manual" or "Partly skilled/Unskilled"), and whether both the ALSPAC-G0 and ALSPAC-G1 participants were residing in a home with a Bristol (BS) postcode at the time of recruitment (by definition this was ‘yes’ for all ALSPAC-G0/G1 pregnancies). The imputation model in both of the separate (ALSPAC-G0/G1 and ALSPAC-G1/G2) generation analyses included all variables used in any of the cross-cohort association analyses specific to that generation listed in Table 3 (e.g. in ALSPAC G0/G1 the prediction model included ALSPAC-G0 (maternal) age and in ALSPAC G1/G2 it included G1 (maternal) age). In addition, as with the combined imputation model described above, both of these separate gestational prediction models included whether the ALSPAC-G0 (for -G0/G1 imputation) or ALSPAC-G1 (for -G1/G2) ever smoked (outside of pregnancy), the ALSPAC-G0 or ALSAPC-G1 mother’s education and their mother’s social class. In the separate imputation model for the ALSPAC-G1/G2 generation we additionally included pregnancy glucose levels (these data were not available for G0/G1 pregnancies) and whether they resided in a Bristol postcode or not (this variable was not included in the G0/G1 imputation model as by definition this was always ‘yes’).

**Table 3.** Comparison of pregnancy, birth and infancy characteristics between pregnancies occurring 1990 to 1992 (parent generation; ALSPAC-G0/G1; restricted to those aged 19–26 years at the birth of their G1 child) and those occurring 2012–2018 (contemporary generation; ALSPAC-G1/G2).

*Continuous outcomes*	G0/G1; pregnancies 1991–1992 (N = 5,287)		G1/G2; pregnancies 2012–2018 (N = 494)				
	N (%) with data	Mean (SD)	N (%) with data	Mean (SD)	Age adjusted difference in mean (95%CI) ^a^ Observed (complete) data	Age adjusted difference in mean (95%CI) ^a^ Combined generation multiple imputed data	Age adjusted difference in mean (95%CI) ^a^ Separate generation multiple imputed data
Age at pregnancy/ delivery (Years)	5287 (100)	23.1 (2.6)	494 (100)	21.7 (2.6)	-1.4 (-1.16, -1.64)	No missing data	No missing data
Pregnancy BMI (Kg/m ^2^) ^b^	2719 (51)	23.9 (4.4)	257 (52)	25.1 (5.7)	1.50 (0.77, 2.23)	1.38 (0.58, 2.18)	1.65 (0.73, 2.58)
Pregnancy cholesterol (mmol/)	2283 (43)	4.9 (1.5)	133 (27)	6.4 (1.2)	1.6 (1.3, 1.8)	1.52 (1.26, 1.79)	1.41 (1/17, 1.65)
Pregnancy haemoglobin (g/dL)	4303 (81)	12.5 (0.9)	285 (58)	12.6 (1.1)	0.08 (-0.06, 0.21)	0.00 (-0.13, 0.12)	-0.03 (-0.18, 0.12)
Pregnancy glucose (mmol/)	NA	NA	132 (27)	5.0 (1.1)	NA	NA	NA
Gestational age (completed weeks) ^e^	5287 (100)	39.4 (2.1)	270 (55)	39.4 (2.0)	-0.01 (-0.26, 0.24)	0.01 (-0.27, 0.29)	0.08 (-0.21, 0.36)
Birthweight (g) ^e^	5217 (99)	3340 (570)	367 (74)	3410 (581)	96 (35, 158)	102 (41, 164)	108 (46, 170)
*Binary outcomes*	G0/G1; pregnancies 1991–1992 (N = 5,287)		G1/G2; pregnancies 2012–2018 (N = 494)				
	N (%) with data	Number with outcome (%)	N (%) with data	Number with outcome (%)	Age adjusted Odds ratio (95%CI) ^a^ Observed (complete) data	Age adjusted odds ratio (95%CI) ^a^ Combined generation multiple imputed data	Age adjusted odds ratio (95%CI) ^a^ Separate generation multiple imputed data
Educated to A-level or higher ^c^	4410 (83)	827 (19)	321 (65)	174 (54)	7.53 (5.85, 9.7)	7.72 (6.00, 9.92)	7.84 (6.08, 10.10)
Smoked cigarettes in pregnancy ^d^	4953 (94)	1948 (39)	210 (43)	33 (16)	0.25 (0.17, 0.36)	0.28 (0.19, 0.40)	0.32 (0.22, 0.45)
Delivered by Caesarean Section ^e^	3245 (61)	453 (14)	274 (55)	53 (19)	1.66 (1.20, 2.28)	1.71 (1.50, 2.53)	1.79 (1.24, 2.58)
Ever breastfed ^d^	3779 (71)	2548 (67)	326 (66)	259 (79)	2.18 (1.65, 2.89)	2.21 (1.66, 2.93)	2.11 (1.58, 2.82)

Results are all from the ALSPAC-G0/ALSPAC-G1 (G0/G1) and ALSPAC-G1/ALSPAC-G2 (G1/G2) mother-offspring pairs. G0 women were recruited in pregnancy between 1990–1992; G1 are the index female offspring from those pregnancies or the female partners of the index male offspring; G2 are their offspring (grandchildren of G0). In both groups only the first pregnancy recruited to the study are included. There was a greater proportion of pregnancies removed from G1/G2 (217 out of 711 (31%) than G0/G1 (111 out of 5398 (2%)) reflecting the fact that G1/G2 is an open cohort recruiting all children to the original ALSPAC-G1 cohort. G1/G2 pregnancies occurred between June 2012 and June 2018. For both G0/G1 and G1/G2 analyses are restricted to women who were aged 19–26 years during their pregnancy (the age range for the majority of the G1 women when they were pregnant with G2 offspring).

^a^ Difference in mean age unadjusted; all other differences in means or odds ratios adjusted for maternal age. In all analyses G0/G1 are the reference category; ^b^ For both G0 and G1 women weight was abstracted from the first antenatal clinic visit; height was self-reported for G0 and measured in the ALSPAC clinic for G1; ^c^ A-levels: Advanced-levels, secondary school exams required for University entrance and some other further education/apprentice schemes taken at age 18. Education data was obtained from self-report for G0 and record linkage for G1; ^d^ Obtained using same questions in G0 and G1; ^e^ Obtained from medical record linkage in both G0 and G1.

In all imputation models we used an augmented regression approach with small weights to prevent perfect prediction for whether ALSPAC-G0 or -G1 ever smoked (outside of pregnancy), as someone who never smoked could not have smoked in pregnancy. This augmented regression approach was also required in the combined imputation model to prevent perfect prediction between Bristol postcode and cohort (as all G0/G1 pregnancies were ‘yes’ to having a BS postcode). This augmented regression approach was necessary as it is not possible to estimate parameters for perfectly predictive variables, and therefore imputation based on these variables is subsequently not possible ^14^. Differences (differences in means or odds ratios) between generations were estimated in each of the 100 imputed datasets and then pooled using Rubin’s rules ^8^.

These preliminary analyses suggest that the current generation of pregnant women (ALSPAC-G1 mothers with their G2 child) are slightly younger, have a higher body mass index and total cholesterol. They also have markedly higher odds of completing education to at least advanced (A)-level secondary school qualifications and markedly lower odds of smoking during pregnancy than their mother’s generation ( Table 3). Their (ALSPAC-G2) children are more likely to be delivered by Caesarean section, have a higher mean birth weight and are more likely to have been breast fed as infants than the ALSPAC-G1 generation. Pregnancy haemoglobin levels and gestational age at delivery are similar between the two generations ( Table 3). We do not have pregnancy glucose levels for the ALSPAC-G0 generation but present these for the ALSPAC-G1 generation in Table 3. The results are consistent when based on observed data or from pooling of multiple imputed datasets irrespective of whether the imputed datasets were generated with ALSPAC-G0/G1 and ALSPAC-G1/G2 combined or done separately in each generation prior to the main analyses.

***Pilot data collection of physical activity and diet***. We piloted the use of a custom made wrist-worn device, which includes a triaxial accelerometer sensor, low-power radio, battery and non-volatile memory module, on 97 mothers ^15^. The motivation was to be able to collect very detailed activity data over extended periods of time with as little inconvenience to the participants as possible. The device stores accelerometer data in non-volatile memory allowing for those data to be retrieved (some time later) over a low-power wireless link. It uses a novel, low power, lossless data compression algorithm to minimise the amount of data transmitted over the radio link, whilst retaining all information captured by the accelerometer. This, combined with the low-power radio, makes it possible to collect very detailed activity data whilst minimising overall device power consumption, reducing the burden of having to keep the device charged ^15^. However, the pilot study found that participants were unlikely to wear this device because its large size meant that they found it less ‘attractive’ than modern smart watches. We are now collecting pregnancy physical activity levels using the Axivity AX3 wrist-worn triaxial accelerometer with a considerably greater uptake than the custom made accelerometer (84% versus 50%), resulting in those who provided valid data being greater for the Axivity AX3 accelerometer (254 (79%) of the 322 eligible) compared with the custom-made system (263 (46%) of the 576 eligible).

We are one of the first studies to explore the feasibility of using a smartphone food-photography application to assess dietary intake in a general population of young pregnant women ^12^. The Remote Food Photography Method (RFPM) collects dietary data using the SmartIntake phone application ^16^. Pregnant ALSPAC-G1 participants (carrying an ALSPAC-G2 fetus) were asked to record 6 days of eating/drinking occasions with this smartphone application. Real-time monitoring and feedback occurred for the first day. This required them to take a photograph before and after each eating/drinking occasion and provide a brief text description of items that are not visible in photos (e.g. butter inside sandwiches). A total of 182 mothers who were recruited at any assessment point during pregnancy were invited to use RFPM and/or an online food diary or recall to collect dietary data for 6 consecutive days. A greater proportion of women agreed to use the online method compared with RFPM (53% vs 22%). However, of those agreeing to use RFPM, more provided data for 4 days or more than those agreeing to complete the online diary or recall assessments (58% vs 29%). Of those using the RFPM, most found installation and set-up (95%), taking photos of meals (70%) and receiving reminders (81%) easy or very easy ^12^.

As the use of expert analysis of food photos is extremely research-resource-intensive, we also completed pilot work comparing portion size and food groups identified by expert analysis with crowd-sourced data (using photographs from non-ALSPAC volunteers). We used 30 photographs of meals. For each of these photos total meal weight was measured using an Mandometer® device (Mikrodidakt AB, Lund, Sweden) and food groups displayed were reviewed by an expert dietitian. In comparison to the measured weight, crowds underestimated meal weight by an average of 63 g (95% level of agreement -299 to 174 g), whereas the dietitian overestimated by 28 g (95% level of agreement -158 to 214 g) ^17^. In further analyses, we found that compared with expert dietician review, crowds varying in size from 5 to 50 people identified food groups in the photos with high specificity (mean 98%) but modest sensitivity (68%) i.e. crowds almost always identified foods in the photo correctly but some foods in photos were missed out, explaining the average underestimation of total meal weight by crowds (unpublished findings).

***Using continuous glucose monitors in unselected prenatal/postnatal women***. We have completed a pilot study of the use of continuous glucose monitors (CGM) in healthy women during pregnancy and postnatally. Women were invited to wear a Medtronic iPro2 CGM on their buttock, abdomen or arm, for 6-days, at up to four time points: in early pregnancy (≤20 weeks gestation), late pregnancy (>28 weeks), and at 6 and 12 months postnatally. A total of 63 women provided 96 CGM assessment (25% response) ^10^. While wearing the device, participants were asked to measure their capillary blood glucose by finger prick 4 times daily to calibrate the system and to record mealtimes in a meal diary. Feedback from participants suggests that the requirement to repeatedly test capillary glucose with a finger prick was a disincentive to using this system and in Summer 2019 we started using a different system that does not require calibration using capillary blood from finger pricks.

We used CGMs to record interstitial glucose ‘continuously’, producing a sequence of measurements for each participant (the interstitial glucose every 5 minutes, over a 24-hour period for a 6-day period). To analyse these data, researchers tend to derive summary variables such as time spent above or below specific levels. To date, a lack of consistency and transparency of precise definitions used for these descriptive characteristics has hindered interpretation, replication and comparison of results across studies ^18^. We have developed an open-source software package (GLU) for deriving a consistent set of summary variables from CGM data ^10^. GLU performs quality control of each CGM sample (e.g. addressing missing data), derives a diverse set of summary variables covering six broad domains, and outputs these measures to the user. We have used GLU with the ALSPAC-G2 pilot data and shown that overall mean glucose levels were very similar (~5 mmol/l) across the four time points, but that this similarity conceals very different patterns of variation, with greater variability during pregnancy (both early and late) than postnatally and more time spent hypoglycaemic during pregnancy than postnatally. Fasting glucose was, on average, higher 12 months postnatally compared with early pregnancy ^10^. We also found that, during pregnancy, higher BMI was associated with higher overall mean glucose levels during both the day and night, higher time spent in hyper-glycaemia during the night and shorter post-prandial time to peak glucose ^10^.

## What are main strengths and weaknesses?

***Main strengths***. ALSPAC-G2 makes the whole ALSPAC resource a unique intergenerational scientific resource for researchers globally. Recruiting ALSPAC-G2 provides a pre-conception cohort with very detailed information on at least one parent from when they were *in utero*. For the parent who was not a member of the original ALSPAC-G1 cohort, extension of our record linkage to primary and secondary health care, and school-based educational assessments, will provide some pre-conceptual data, and for those with a subsequent child there will be pre-conceptual data on the second and subsequent children. ALSPAC-G2 also has more data on fathers than in most pregnancy/birth cohorts. Once all of the next generation have been born, we will hopefully have a cohort that includes multiple sibling groups and other extended family relationships that can be exploited to improve causal inference ^5^, as well as understanding how family relationships impact health and wellbeing. We will also have a cohort (the original ALSPAC-G1) on whom we have prospective data that can be used to describe subgroups of those who choose to start a family at a relatively young age, compared with those who start their family at older ages, those who choose to remain childless and those who are childless but not through choice. It will be possible to explore factors related to these choices and how they relate to future health and wellbeing of the parents/adults (G1) and their children (G2). No other study that we are aware of will have such prospective data.

An additional strength is that we will use ALSPAC-G2 participants as a control cohort for local and national clinical cohorts, including the on-going national cleft lip and palate cohort ( http://www.bris.ac.uk/dental/cleft-collective/professionals/), and newly planned Bristol IVF ( https://www.bristolbrc.nihr.ac.uk/our-research/perinatal-and-reproductive-health/improving-outcomes-for-in-vitro-fertilisation-ivf/ivf-study/) and congenital heart disease cohorts (currently funded with recruitment to start soon but no reference or website available). We have demonstrated the value of ALSPAC-G2 for piloting and testing the feasibility of novel data collection methods and work closely with other international birth cohorts to share best practice, protocols and replicate, and where appropriate pool data.

***Main limitations***. Currently the number of ALSPAC-G2 participants is relatively small and whilst we have been able to identify large between generation differences, such as the difference in antenatal depression symptoms and smoking during pregnancy, for more modest but potentially important differences it may be some time before these can be precisely estimated. That said, participant numbers are increasing as the G1 participants approach the current mean age for a first pregnancy in the UK (29 years) ^19^.

Other key limitations fall into one of three areas: sources of selection bias; the representativeness of each generation to a target population; and limitations related to different data collection approaches and change in these over time. These are discussed in detail below.

***Selection bias***. There are a number of sources of potential selection bias in ALSPAC-G2 ^20^. Some of these, such as incomplete recruitment and loss to follow-up, are common to most prospective cohort studies, whereas others, such as the current recruitment at a relatively young age and the focus of analyses in cross-generational effects, are more specific to ALSPAC-G2. Below we describe some of the key potential sources of selection bias, general methods that might be used to mitigate against some of the selection mechanisms, and data and analytical approaches that could be used to explore and control for potential selection bias. It is important to note that appropriate methods for exploring and controlling for selection bias are specific to the research question and the analyses being used to address that question. Furthermore, ALSPAC is a resource for the global scientific community to use and we do not dictate how scientists using this resource complete their analyses. Whilst we make suggestions here that might be used to explore selection bias, researchers must decide how they want to approach this for the specific questions they are exploring with these data.

Our response is 56% of those invited (53% overall) and respondents are more likely to be those already engaged with ALSPAC as indicated by being more likely to have participated in the two most recent clinic assessments for all ALSPAC-G1 participants. Once recruited a high proportion remain actively engaged with follow-up, though the extent to which this will continue as the cohort ages is unknown. Currently, ALSPAC-G2 are children being born to relatively young parents (mean 22 years (range 17–26)); though the age range of parents will increase with continued follow-up. Whilst cross-generational comparisons that have already been published and/or presented in this paper are age matched, it is possible that the factors that influence starting a family at a relatively young age differ across generations. If these factors are unknown or unmeasured (and therefore cannot be controlled for) there may be bias in the cross generational comparisons. This may be further complicated by secular trends in some of these factors. For example, preliminary analyses presented here suggest that age matched ALSPAC-G1 mothers with an ALSPAC-G2 child are more educated than were their ALSPAC-G0 mother’s generation when recruited during their pregnancy ~30years ago. This difference may be due to true differences between UK pregnant women across generations. For example, there may have been a reduction in the stigma attached to becoming pregnant at a young age and hence a reduction in the association of education/socioeconomic position with it. ALSPAC-G1 mothers may also be truly more educated than their parent’s generation as a result of changes in education policy and secular trends related to that policy. Over the last 20–30 years there has been a doubling of the proportion of people obtaining a University degree in the UK, stimulated by policy changes, including previous further education establishments becoming Universities in the 1990s. Alternatively these cross-generational differences in education could be due to selection bias. This is supported by our finding that those ALSPAC-G1 parents recruited as pregnant women or parents to ALSPAC-G2 are more engaged with the study than those not recruited ( Table 2), and that ALSPAC-G1 participants who are more engaged with the study are on average more educated than those less engaged ^1, 2^.

At the time we began recruitment, ALSPAC-G1 participants were aged 19–21 years and some had already become parents. Thus, whilst the cohort is currently of relatively young parents, we do not have detailed pregnancy data on those who became pregnant before 19-years. To maximise participation of all ALSPAC-G2 we will continue to recruit participants at different ages. For those ALSPAC-G2 participants recruited after birth, either because they were born prior to when we began recruitment or because their parents only decided to join ALSPAC-G2 when their child/children were older, their mother’s pregnancy information and their birth/infancy information will be limited to what we can obtain from record linkage. With the extensive linkage that is now possible, including primary-, secondary- and community health care, education and environmental linkage we will still have a lot of information on these pregnancies and the resulting infants. Furthermore, the proportion recruited in pregnancy has increased over time and once a family have joined ALSPAC-G2, subsequent children are mostly recruited during their mother’s pregnancy.

To mitigate against some of these sources of bias we aim to minimise participant burden by using record linkage and remote data collection as much as possible. The ability to do this is supported by the existence of a National Health Service in the UK and large-scale initiatives across UK research funders for supporting ethical and appropriate access to anonymised health, environmental, and socioeconomic data. Linked data can also be used for describing the likely nature of any selection bias and informing sensitivity analyses.

Both existing research and record linkage data, including on grandparents and parents (ALSPAC-G0 and -G1), can be used to explore potential selection bias from any of the sources described above, and to inform sensitivity analyses to explore the extent of bias that might occur for any given analyses ^20^. In this section we describe some of these approaches in general terms, as it is important to note that which methods are most appropriate to use will depend on the specific research questions being addressed and the likely source of selection bias ^21, 22^. Furthermore, ALSPAC data are available for scientists across the world to use. Whilst we highlight potential sources of selection bias in this cohort profile and will do so through conference presentations and the study website, we cannot dictate how other scientists approach these issues when using the study data. We will encourage others to explore issues of selection bias when using ALSPAC-G2 data and make details of their methods open access (including via linked Wellcome Open Research publications).

In our work we have found directed acyclic graphs ^21^ useful to illustrate specific sources of selection bias and their assumptions of any methods used to explore and control for these (see for example references 20– 24). Selection bias results from missing data (for example, missing data due to not being recruited, being lost to follow-up or missing data on factors influencing different fertility practices across generations) thus methods for dealing with missing data are appropriately used to explore and control for selection bias. Publication reporting guidelines, such as Strengthening The Reporting of Observational studies in Epidemiology (STROBE; https://strobe-statement.org/index.php?id=strobe-home) require authors to describe the extent of missing data and how potential bias from that has been explored and we strongly encourage users of ALSPAC to adhere to those reporting guidelines. Such reporting guidelines do not recommend specific approaches, e.g. for dealing with missing data, just that there is clarity about the issue. A common approach to dealing with missing data is complete case analyses (i.e. in which only participants with complete data on exposure, outcome and all covariables included in the main analyses are included in analyses). In general, this is expected to be unbiased if missingness is not related to the outcome in the main analysis model conditioned on exposure and all covariables included in the main analyses ^23^. Other commonly used methods for exploring and mitigating against selection bias include multiple imputation and inverse probability weighting, both of which assume that data are missing at random (MAR, missingness depends on observed data only ^23^). When analysing repeat measurements, e.g. trajectories of change in BMI or cognitive function, all participants with at least one value (of the variable that has been assessed repeatedly) can be included in the main analyses under a MAR assumption. Ideally, one would want to use more than one method, with differing assumptions, for exploring selection bias (bias due to missing data). In the preliminary cross-generational analyses presented here we compared results from complete case analyses to those using multiple imputation (including using two different approaches to the latter) and found similar results across all three approaches which increases confidence in these not being biased.

In general, research data that have been specifically collected as part of a study and linked data have complementary characteristics for exploring selection bias under the MAR assumption. Specific research data might predict missing values more strongly because it will have been collected at a similar time to variables with missing data (e.g. in sweeps of data collection) and will include some repeat collections of data obtained using the same methods across time. Such data are also likely to be obtained more extensively (e.g. several domains of socioeconomic position) and more precisely (e.g. DEXA scan total and body region fat and lean mass). However, specific research data may have more missingness due to loss to follow-up or some measures not being completed by some participants. Linked data is often available on all (or a large proportion) of participants. All generations of ALSPAC have both repeatedly assessed research and record linked data on many variables (see 1, 2 and Figure 3), which considerably enhance the possibility for exploring and controlling for potential selection bias. Currently, ALSPAC-G0 and ALSPAC-G1 have been linked to obstetric-, primary-, secondary- and community health data, mortality and migration records and education data. Recent geocoding has also provided linkage to detailed environmental and area socioeconomic data ^25^. Similar linkage for ALSPAC-G2 and the new (to ALSPAC) partners of existing ALSPAC-G1 is on-going and will be regularly updated. These data can be informative for methods assuming data are MAR (e.g. 22) and also where selection (missing information) is assumed to be missing not at random (i.e. where bias can remain even after taking into account observed predictors of missingness) ^24^. For example, using linked education data as proxy for IQ in ALSPAC-G1 it has been shown that methods assuming data were missing at random underestimated the strength of the association in comparison to the method assuming missing not at random ^24^. In addition to linked data, genome-wide genetic data, which is available on a high proportion of ALSPAC-G0 and ALSPAC-G1 participants and will be on ALSPAC-G2 and the newly recruited partners of ALSPAC-G1, can be useful in exploring the nature of missing data ^26^.

Whilst we ^22– 24^, and others, (e.g. 27) have used the methods described above extensively in ALSPAC-G0 and ALSPAC-G1, their use for cross-generational analyses, in particular where the inclusion of siblings varies considerably across generations, has not yet been extensively explored. As demonstrated with ALSPAC-G2, such cross-generational analyses by necessity either restrict analyses to a narrow age range of parents in one generation (e.g. if we were to only continue to recruit ALSPAC-G2 for the next 5-years) or have participants recruited over a long period of years in one generation (as we hope to do with ALSPAC-G2). In the cross-generational analyses presented here we have been careful to describe the results as preliminary and illustrative. We undertook complete case analyses and used multiple imputation to explore potential selection due to missing data in the cross-generational comparisons presented here ( Table 3). We used two approaches to multiple imputation, one including both generations in the same imputation analyses and one that separated the G0/G1 from the G1/G2 generations for the initial imputation. We found results were very similar across all three approaches. However, for simplicity, in these illustrative analyses we restricted the maternal age range in both generations to that of the ALSPAC-G1/G2 participants and to just one pregnancy per mother in each generation. This resulted in more of the G1/G2 pregnancies being excluded from analyses than G0/G1 pregnancies, which could have introduced bias that is not addressed in our multiple imputation analyses. In future research we plan to develop and use methods that can more fully explore the sources of selection bias in cross-generational analyses. This includes methods that combine multiple imputation and other missing data methods with methods that appropriately take account of the complex multi-level structure of the data (i.e. repeat pregnancies within women across generations and potentially including complex family structures of siblings, cousins, nieces, nephews and so on). These methods and related analysis code will be disseminated widely via the ALSPAC website and via links to this cohort profile. We hope that other researchers will also use the unique multi-generational data of ALSPAC for exploring the likely impact of selection bias on this and other human multigeneration cohorts.

***Representativeness of different generations to the target population***. The original women recruited to ALSPAC (i.e. ALSPAC-G0) had similar socioeconomic characteristics and ethnic backgrounds to those of the population of the former county of Avon (South West region of England) ^2^. They were more affluent and more likely to be White European than the whole of the UK in the early 1990s, which reflects the difference between the population in the South West of England where the ALSAPC resource is based and the rest of the UK ^2^. At age 16-years their children (ALSPAC-G1 – i.e. the parents of ALSPAC-G2) had somewhat higher school test scores than the national average, with this difference increasing in sub-samples based on increasing loss to follow-up ^1^. As recruitment of ALSPAC-G2 has been higher for those whose ALSPAC-G1 parents were most engaged with the study it is possible that over time ALSPAC-G2 will show greater differences to national averages. We will monitor that through the extensive record linkage, including data on education. Whilst selection bias and representativeness are sometimes treated as if they reflect the same concept, here we consider that lack of representativeness to the target population may bias estimates of prevalence but does not necessarily influence association or causal effect estimates. There have been considerable debates on this topic (see for example 28– 33), with evidence that representativeness is not necessary to obtain unbiased estimates of association or effect ^28– 32^, though this will depend on the extent of selection into a study and the availability of data to predict this ^20, 33^. In this regard a key strength of ALSPAC-G2 is the existence of extensive data on previous generations and linkage to national data across all generations ^33^.

With respect to analyses that might only be done within the ALSPAC-G2 cohort currently, we would consider association or causal effect estimates to generalise to couples becoming pregnant at a relatively young age. This would be consistent with other pregnancy studies that recruit at younger ages and/or only recruit nulliparous women (such as 34, 35). Once we have had the opportunity to invite all of ALSPAC-G2 we will have a unique study in which to explore the relationships of age at which couples become pregnant/parents across a wide age range within a relatively homogeneous cohort in terms of date and place of birth.

***Limitations related to data collection approaches and their change over time***. There are issues related to which measurement tools to use and potential differences in measurement error and/or the specificity of measurements across generations for all types of data, from biomarker through to socioeconomic and from specific research collected to record linkage data. We describe these issues and our current approach to them below.

Having an open cohort raises questions about when to complete assays on stored samples—for example, should we wait until we have 500, 1000, or some higher number of cord-blood samples before we assay DNA methylation on them? Currently, we invite enquiries about using the biosamples and will manage these on a case-by-case basis taking account of participant numbers with a given sample, risk of disclosure and assay/processing features such as the extent of ‘batch’ effects. Our aim is to ensure the resource provides the best data for the widest group of scientists. We have considerable experience of obtaining and analysing multi-‘omic biomarker data and are aware that ‘batch-effects’ vary by analysis type. For the quantified NMR metabolomics used in ALSPAC-G0 and -G1 and many other cohorts, batch effects are minimal ^36^. Batch effects from mass spectrometry (MS) metabolomics can be minimised by repeat normalisation across batches as assays are repeated in new cohort members and for chip based analyses for example, with epigenomic (DNA methylation) data adjusting for technical factors can minimise differences across batches. Furthermore, collaborative research shows considerable consistency across cohorts, despite MS metabolomic (e.g. 37) and DNA methylation data (e.g. 38) having clearly been obtained in different batches between studies included in those collaborations.

There are important considerations about whether we should use older data collection tools, such as those used to assess mental health and lifestyle / behaviours in previous ALSPAC generations (with the advantage that direct comparisons across generations can be made), or more contemporary tools that might be considered more valid and allow comparisons with other contemporary cohorts. Currently, we have mostly used similar tools to those used in the previous generation, but over the next 12–24 months we will undertake workshops with experts in relevant fields to explore whether there are some measures for which we should be using more contemporary tools.

Differences across generations on characteristics obtained through record linkage may be influenced by changes in how disease outcomes are classified over time, changes in policy (for example the recent increase in the minimal legal age for leaving education in the UK) and the methods used to obtain information in these data systems, which are primarily used for administrative or policy purposes rather than research. The data management systems in ALSPAC, together with national initiatives, will ensure we remain aware of such changes and we will provide guidance to users of the resource about their potential impact on ALSPAC data.

We anticipate that most ALSPAC-G2 participants will continue to be born up to the early 2040s (though as some men continue to father children into old age, and we recruit all children, including adopted and ‘step’-children, some ALSPAC-G2 children may be recruited even later than this). By the 2040s there will also be ALSPAC-G3 and -G4 participants. Continuing to recruit these participants and generations adds considerable value to the existing ALSPAC resource for the whole scientific community. It has the potential to uniquely address important research questions related to how development and adverse health outcomes are transmitted across generations and how we might intervene to maximise population health. However, it may be difficult to continue to obtain the necessary funds for continued recruitment of, and data collection from, multiple generations, and it may also be difficult to retain participant engagement over prolonged periods of time. To address this, we are pursuing more extensive record linkage and novel remote minimally intrusive data capture, such as via smart-watches ^39^ and sensors for biomarkers ^10^, to provide efficient (and accurate) data collection. The novel data sensors we are currently using are likely to be replaced by new systems in the future. We are committed to continue piloting novel data collection methods in ALSPAC-G2 and calibrate them against approaches that have previously been used in ALSPAC-G2 and -G0 and -G1. As an example, we have recently changed from a continuous glucose monitoring system that required participants to complete four finger prick tests of their capillary blood glucose per day, to one that does not have this requirement. We have directly compared these two systems in (non-ALSPAC) volunteers who wore both monitors at the same time to establish consistency in traces between them. We intend to learn from our increasing experience of harmonising data collected in many different ways across cohorts in several collaborations. With respect to birth cohorts (including all generations of ALSPAC), this is currently being undertaken for substantial amounts of data in the Horizon2020 funded LifeCycle collaboration ( https://lifecycle-project.eu/), which will, together with other collaborative efforts, provide the foundations for continuing this with different data collection tools over coming years and decades in ALSPAC-G2.

The fact that this is a cohort in which at least one parent has been actively engaged with the study for over 20-years may mean that they have ‘learnt’ how to respond to some research questions, such as those related to mental health, or complete some assessments, such as cognitive function tests ^40^. A ‘learning effect’ will be unlikely for direct measurements such as weight, height and biosample assays. The ALSPAC-G2 participants are mostly born in the South West of England (as were all of the original ALSPAC-G1 participants) and are mostly of White European origin. This has some advantages for exploring between generational differences, but we acknowledge replication of findings from ALSPAC-G2 with other more diverse cohorts will be important.

To conclude, ALSPAC-G2 provides a unique resource for exploring how health and wellbeing are transmitted across generations. It is a complex study with multiple sources of potential selection bias. We (and hopefully other researchers) will use these data for applied research and also to develop valid methods for appropriately exploring and controlling for selection bias and for analysing the complex structure of these family data. This will enhance the value of ALSPAC-G2 and also be of considerable benefit to other family based cohorts, in particular those that are multi-generational.

## Data availability

The ALSPAC data management plan ( http://www.bristol.ac.uk/alspac/researchers/data-access/documents/alspac-data-management-plan.pdf) describes in detail the policy regarding data sharing, which is through a system of managed open access. The steps below highlight how to apply for access to the data included in this paper and all other ALSPAC data.

1. Please read the ALSPAC access policy (PDF, 627kB) which describes the process of accessing the data and samples in detail, and outlines the costs associated with doing so.
2. You may also find it useful to browse the fully searchable ALSPAC research proposals database, which lists all research projects that have been approved since April 2011.
3. Please submit your research proposal for consideration by the ALSPAC Executive Committee. You will receive a response within 10 working days to advise you whether your proposal has been approved.

If you have any questions about accessing data, please email alspac-data@bristol.ac.uk.

We are very keen for ALSPAC-G2 data to be enhanced and used by external collaborators and are happy for email queries to D.A. Lawlor ( d.a.lawlor@bristol.ac.uk) or M. Lewcock ( Melanie.Lewcock@bristol.ac.uk).

Please note that the study website contains details of all the data that is available through a fully searchable data dictionary ( http://www.bris.ac.uk/alspac/researchers/data-access/data-dictionary/).

### Extended data

Details of all of the measurements that are currently being collected on ASPAC-G2 participants and their parents (ALSPAC-G1), including the available stored biosamples, alongside the participant information sheet that we developed with ALSPAC-G2 children for use from age ~9-years, can be accessed on the Open Science Framework. DOI: https://doi.org/10.17605/OSF.IO/4APU8 ^9^.
