# Supplementary material for: The second generation of The Avon Longitudinal Study of Parents and Children (ALSPAC-G2): a cohort profile
Source: Wellcome Open Res. 2019 Dec 16;4:36. Originally published 2019 Feb 20. [Version 2] doi: 10.12688/wellcomeopenres.15087.2 (PMC6971848; doi:10.12688/wellcomeopenres.15087.2)
Supplement: Peer review history

# Peer review: Reviewer response for version 2

**DOI:** 10.21956/wellcomeopenres.17079.r37442

**Published:** 2019-12-20

**Authors:** Susan M. Morton

**Affiliations:** 1 Growing Up in New Zealand, School of Population Health, University of Auckland, Auckland, New Zealand

I have no additional comments to make. The authors have fully and appropriately addressed the comments I provided. I am very grateful for the careful attention to the comments made and the amendments made to the paper as a result. It is excellent to see this article published.

I confirm that I have read this submission and believe that I have an appropriate level of expertise to confirm that it is of an acceptable scientific standard.

# Peer review: Reviewer response for version 1

**DOI:** 10.21956/wellcomeopenres.16460.r34960

**Published:** 2019-05-02

**Authors:** Susan M. Morton

**Affiliations:** 1 Growing Up in New Zealand, School of Population Health, University of Auckland, Auckland, New Zealand

This is an exciting research initiative to create a multi-generational data resource building on an existing longitudinal birth cohort study that has already produced some excellent research results. The aims of the research are appropriate.

 However the aim to recruit all G2 births, while scientifically rational, is logistically challenging and introduces some methodological issues and limitations that currently appear to only be partially addressed in the paper. In particular: 

- A challenge with multi-generational cohorts (regardless of the detail and value of their data collections) is which of the G0 through G2 populations is the index generation - and then what is the generalisability of the others. It seems in this case that G1 is the cohort which is clearly generalisable to a "population of interest" and that G0 and G2 will always be relevant only relative to that generation. This becomes especially problematic when trying to answer research questions that are relevant to G2. Technically it may take three plus decades to collect all G2 information. This matters because those who do reproduce from G1 (male or female) at an early age (currently up to 28?) are systematically different from those who reproduce later even in terms of parental age at first birth. This bias is confounded further by fertility rates and number of offspring in a G2 cohort that aims to capture all offspring. Hence any full analyses to address the overarching research aims with respect to G2 (and G! parenthood) cannot be properly conducted until all reproductive events have concluded. This limitation is not adequately addressed in the current text. 
- Furthermore, this Open G2 cohort (so described) are currently having data collected using bespoke digital tools, including mobile devices - will this still be relevant for G2 children born in two decades time? This needs further explanation/discussion in the text.
- The methodology to deal with the comparability of measurement taken over two or more decades on the G2 (and G1 parents) also needs to be better explained.
- Also of note currently is the low rate of agreement to participate in the G1/G2 collection  - 52% based on 93% invited and 56% agreed. Statistical imputation makes sense but it is not clear how the bias in those delivering G2 offspring and the determinants of this can compensate for the fertility biases - more clarity in this regard would assist.
- It is clear that analyses to date have focussed on age specific analyses  - which seems appropriate - it would be helpful to see further detail around how this will be managed as births accumulate and what research questions can be addressed in the immediate, 5 and 10 year future for example.
- While creating a comparison cohort would be a useful adjunct to the recruitment of the G2 cohort this seems an unlikely reality in the short term given the bias in fertility rates and parental age at birth (as above) - if this is feasible then further details are required.

On the positive side making the multigenerational data resource available is excellent and the Data Access process is clearly articulated. Over the next two or more decades this will create a unique three generation resource - the challenge to users will be how to utilise appropriate methodologies to deal with the complexities of and delays in data capture over time.

I confirm that I have read this submission and believe that I have an appropriate level of expertise to confirm that it is of an acceptable scientific standard, however I have significant reservations, as outlined above.

# Response

**Published:** 2019-11-14

**Authors:** Deborah Lawlor

**Affiliations:** University of Bristol, UK

This is an exciting research initiative to create a multi-generational data resource building on an existing longitudinal birth cohort study that has already produced some excellent research results. The aims of the research are appropriate.

However, the aim to recruit all G2 births, while scientifically rational, is logistically challenging and introduces some methodological issues and limitations that currently appear to only be partially addressed in the paper.

In particular:

 

- A challenge with multi-generational cohorts (regardless of the detail and value of their data collections) is which of the G0 through G2 populations is the index generation - and then what is the generalisability of the others. It seems in this case that G1 is the cohort which is clearly generalisable to a "population of interest" and that G0 and G2 will always be relevant only relative to that generation. This becomes especially problematic when trying to answer research questions that are relevant to G2. Technically it may take three plus decades to collect all G2 information. This matters because those who do reproduce from G1 (male or female) at an early age (currently up to 28?) are systematically different from those who reproduce later even in terms of parental age at first birth. This bias is confounded further by fertility rates and number of offspring in a G2 cohort that aims to capture all offspring. Hence any full analyses to address the overarching research aims with respect to G2 (and G! parenthood) cannot be properly conducted until all reproductive events have concluded. This limitation is not adequately addressed in the current text.

**Response**

We have not emphasised an index generation as ALSPAC is and always has been a resource for the global research community. As with other such UK National cohorts we do not dictate how other scientists use the resource (other than ensuring the resource is always used within the ethical and legal framework specific to ALSPAC and as governed by all relevant research governance authorities). Whilst we see considerable benefit in doing research that uses more than one generation the resource can and will continue to use single generations. That said, this cohort profile and the previous ALSPAC profile is hopefully a valuable resource for the research community that should highlight issues that should be considered by anyone using the data. We have not specified an index generation in the revised paper as we are not sure it is possible to say that should be G0 or G1. The resource has evolved since it was started nearly 30 years ago and the addition of G2 is just one recent example of that evolution. For example, the study was originally called the Avon Longitudinal Study of Pregnancy And Childhood (see e.g. https://www.ncbi.nlm.nih.gov/pubmed/8527273) giving equal prominence to G0 and G1. As a scientific case for longitudinal follow-up provided support to continue the study the name was changed to Avon Longitudinal Parents and Children ( https://academic.oup.com/ije/article/42/1/111/694290/). We have however, added a section to the part of the discussion on strengths and limitations on ‘Representativeness of different generations to the target population’:

*“ **Representativeness of different generations to the target population***

*The original women recruited to ALSPAC (i.e. ALSPAC-G0) had similar socioeconomic characteristics and ethnic backgrounds to those of the population of the former county of Avon (South West region of England). ^2^ They were more affluent and more likely to be White European than the whole of the UK in the early 1990s, which reflects the difference between the population in the South West of England where the ALSAPC resource is based and the rest of the UK. ^2^ At age 16-years their children (ALSPAC-G1 – i.e. the parents of ALSPAC-G2) had somewhat higher school test scores than the national average, with this difference increasing in sub-samples based on increasing loss to follow-up. ^1 ^As recruitment of ALSPAC-G2 has been higher for those whose ALSPAC-G1 parents were most engaged with the study it is possible that over time ALSPAC-G2 will show greater differences to national averages. We will monitor that through the extensive record linkage, including data on education. Whilst selection bias and representativeness are sometimes treated as if they reflect the same concept, here we consider that lack of representativeness to the target population may bias estimates of prevalence but does not necessarily influence association or causal effect estimates. There have been considerable debates on this topic (see for example ^28-33^), with evidence that representativeness is not necessary to obtain unbiased estimates of association or effect, ^28-32^ though this will depend on the extent of selection into a study and the availability of data to predict this. ^20,33^ In this regard a key strength of ALSPAC-G2 is the existence of extensive data on previous generations and linkage to national data across all generations. ^33^*

*With respect to analyses that might only be done within the ALSPAC-G2 cohort currently, we would consider association or causal effect estimates to generalise to couples becoming pregnant at a relatively young age. This would be consistent with other pregnancy studies that recruit at younger ages and/or only recruit nulliparous women (such as ^34,35^). Once we have had the opportunity to invite all of ALSPAC-G2 we will have a unique study in which to explore the relationships of age at which couples become pregnant/parents across a wide age range within a relatively homogeneous cohort in terms of date and place of birth. (* **Discussion section of the paper page 27-28 of clean version** *)*

- Furthermore, this Open G2 cohort (so described) are currently having data collected using bespoke digital tools, including mobile devices - will this still be relevant for G2 children born in two decades time? This needs further explanation/discussion in the text. The methodology to deal with the comparability of measurement taken over two or more decades on the G2 (and G1 parents) also needs to be better explained.

**Response**

We agree this is an important point across all of the generations and have now extended our discussion of this with a subheading that highlights the issue of harmonising repeat measurements across the generations:

***“Limitations related to data collection approaches and their change over time***

*There are issues related to which measurement tools to use and potential differences in measurement error and/or the specificity of measurements across generations for all types of data, from biomarker through to socioeconomic and from specific research collected to record linkage data. We describe these issues and our current approach to them below.*

*Having an open cohort raises questions about when to complete assays on stored samples— for example, should we wait until we have 500, 1000, or some higher number of cord-blood samples before we assay DNA methylation on them? Currently, we invite enquiries about using the biosamples and will manage these on a case-by-case basis taking account of participant numbers with a given sample, risk of disclosure and assay/processing features such as the extent of ‘batch’ effects. Our aim is to ensure the resource provides the best data for the widest group of scientists. We have considerable experience of obtaining and analysing multi-‘omic biomarker data and are aware that ‘batch-effects’ vary by analysis type. For the quantified NMR metabolomics used in ALSPAC-G0 and -G1 and many other cohorts, batch effects are minimal. ^36^ Batch effects from mass spectrometry (MS) metabolomics can be minimised by repeat normalisation across batches as assays are repeated in new cohort members and for chip based analyses for example with epigenomic (DNA methylation) adjusting for technical factors can minimise differences across batches. Furthermore, collaborative research shows considerable consistency across cohorts, despite MS metabolomic (e.g. ^37^) and DNA methylation data (e.g. ^38^) having clearly been obtained in different batches between studies included in those collaborations.*

*There are important considerations about whether we should use older data collection tools, such as those used to assess mental health and lifestyle / behaviours in previous ALSPAC generations (with the advantage that direct comparisons across generations can be made), or more contemporary tools that might be considered more valid and allow comparisons with other contemporary cohorts. Currently, we have mostly used similar tools to those used in the previous generation, but over the next 12–24 months we will undertake workshops with experts in relevant fields to explore whether there are some measures for which we should be using more contemporary tools.*

*Differences across generations on characteristics obtained through record linkage may be influenced by changes in how disease outcomes are classified over time, changes in policy (for example the recent increase in the minimal legal age for leaving education in the UK) and the methods used to obtain information in these data systems, which are primarily used for administrative or policy purposes rather than research. The data management systems in ALSPAC, together with national initiatives, will ensure we remain aware of such changes and we will provide guidance to users of the resource about their potential impact on ALSPAC data.*

*We anticipate that most ALSPAC-G2 participants will continue to be born up to the early 2040s (though as some men continue to father children into old age, and we recruit all children, including adopted and ‘step’-children, some ALSPAC-G2 children may be recruited even later than this). By the 2040s there will also be ALSPAC-G3 and -G4 participants. Continuing to recruit these participants and generations adds considerable value to the existing ALSPAC resource for the whole scientific community. It has the potential to uniquely address important research questions related to how development and adverse health outcomes are transmitted across generations and how we might intervene to maximise population health. However, it may be difficult to continue to obtain the necessary funds for continued recruitment of, and data collection from, multiple generations, and it may also be difficult to retain participant engagement over prolonged periods of time. To address this, we are pursuing more extensive record linkage and novel remote minimally intrusive data capture, such as via smart-watches* *^38^* *and sensors for biomarkers* *^10^* *, to provide efficient (and accurate) data collection. The novel data sensors we are currently using are likely to be replaced by new systems in the future. We are committed to continue piloting novel data collection methods in ALSPAC-G2 and calibrate them against approaches that have previously been used in ALSPAC-G2 and -G0 and -G1. As an example, we have recently changed from a continuous glucose monitoring system that required participants to complete four finger prick tests of their capillary blood glucose per day, to one that does not have this requirement. We have directly compared these two systems in (non-ALSPAC) volunteers who wore both monitors at the same time to establish consistency in traces between them. We intend to learn from our increasing experience of harmonising data collected in many different ways across cohorts in several collaborations. With respect to birth cohorts (including all generations of ALSPAC), this is currently being undertaken for substantial amounts of data in the Horizon2020 funded LifeCycle collaboration (* *https://lifecycle-project.eu/* *), which will, together with other collaborative efforts, provide the foundations for continuing this with different data collection tools over coming years and decades in ALSPAC-G2.*

*The fact that this is a cohort in which at least one parent has been actively engaged with the study for over 20-years may mean that they have ‘learnt’ how to respond to some research questions, such as those related to mental health, or complete some assessments, such as cognitive function tests ^39 ^. A ‘learning effect’ will be unlikely for direct measurements such as weight, height and biosample assays. The ALSPAC-G2 participants are mostly born in the South West of England (as were all of the original ALSPAC-G1 participants) and are mostly of White European origin. This has some advantages for exploring between generational differences, but we acknowledge replication of findings from ALSPAC-G2 with other more diverse cohorts will be important* *.” *( **Discussion section Page 28-29**)

- Also of note currently is the low rate of agreement to participate in the G1/G2 collection - 52%  based on 93% invited and 56% agreed. Statistical imputation makes sense but it is not clear how the bias in those delivering G2 offspring and the determinants of this can compensate for the fertility biases - more clarity in this regard would assist.  

**Response **We have considerably extended the section on selection bias in the discussion. In relation to this point, this includes the ability to use record linkage and existing research data to inform imputation models and methods for dealing with selection bias.

The updated section is copied in full below:

***“Selection bias***

*There are a number of sources of potential selection bias in ALSPAC-G2* *^20^* *. Some of these, such as incomplete recruitment and loss to follow-up, are common to most prospective cohort studies, whereas others, such as the current recruitment at a relatively young age and the focus of analyses in cross-generational effects, are more specific to ALSPAC-G2. Below we describe some of the key potential sources of selection bias, general methods that might be used to mitigate against some of the selection mechanisms, and data and analytical approaches that could be used to explore and control for potential selection bias. It is important to note that appropriate methods for exploring and controlling for selection bias are specific to the analyses being undertaken. Furthermore, ALSPAC is a resource for the global scientific community to use and we do not dictate how scientists using this resource complete their analyses. Whilst we make suggestions here that might be used to explore selection bias, researchers must decide how they want to approach this for the specific questions they are exploring with these data.*

*Our response is 56% of those invited (53% overall) and respondents are more likely to be those already engaged with ALSPAC as indicated by being more likely to have participated in the two most recent clinic assessments for all ALSPAC-G1 participants. Once recruited a high proportion remain actively engaged with follow-up, though the extent to which this will continue as the cohort ages is unknown. Currently, ALSPAC-G2 are children being born to relatively young parents (mean 22 years (range 17-26)); though the age range of parents will increase with continued follow-up. Whilst cross-generational comparisons that have already been published and/or presented in this paper are age matched, it is possible that the factors that influence starting a family at a relatively young age differ across generations. If these factors are unknown or unmeasured (and therefore cannot be controlled for) there may be bias in the cross generational comparisons. This may be further complicated by secular trends in some of these factors. For example, preliminary analyses presented here suggest that age matched ALSPAC-G1 mothers with an ALSPAC-G2 child are more educated than were their ALSPAC-G0 mother’s generation when recruited during their pregnancy ~30years ago. This difference may be due to true differences between UK pregnant women across generations. For example, there may have been a reduction in the stigma attached to becoming pregnant at a young age and hence a reduction in the association of education/socioeconomic position with it. ALSPAC-G1 mothers may also be truly more educated than their parent’s generation as a result of changes in education policy and secular trends related to that policy. Over the last 20-30 years there has been a doubling of the proportion of people obtaining a University degree in the UK, stimulated by policy changes, including previous further education establishments becoming Universities in the 1990s. Alternatively these cross-generational differences in education could be due to selection bias. This is supported by our finding that those ALSPAC-G1 parents recruited as pregnant women or parents to ALSPAC-G2 are more engaged with the study than those not recruited (Table 2), and that ALSPAC-G1 participants who are more engaged with the study are on average more educated than those less engaged ^1,2^.*

*At the time we began recruitment, ALSPAC-G1 participants were aged 19–21 years and some had already become parents. Thus, whilst the cohort is currently of relatively young parents, we do not have detailed pregnancy data on those who became pregnant before 19-years. To maximise participation of all ALSPAC-G2 we will continue to recruit participants at different ages. Forthose ALSPAC-G2 participants recruited after birth, either because they were born prior to when we began recruitment or because their parents only decided to join ALSPAC-G2 when their child/children were older, their mother’s pregnancy information and their birth/infancy information will be limited to what we can obtain from record linkage. With the extensive linkage that is now possible, including primary-, secondary- and community health care, education and environmental linkage we will still have a lot of information on these pregnancies and the resulting infants. Furthermore, the proportion recruited in pregnancy has increased over time and once a family have joined ALSPAC-G2, subsequent children are mostly recruited during their mother’s pregnancy.*

*To mitigate against some of these sources of bias we aim to minimise participant burden by using record linkage and remote data collection as much as possible. The ability to do this is supported by the existence of a National Health Service in the UK and large-scale initiatives across UK research funders for supporting ethical and appropriate access to anonymised health, environmental, and socioeconomic data. Linked data can also be used for describing the likely nature of any selection bias and informing sensitivity analyses.*

*Both existing research and record linkage data, including on grandparents and parents (ALSPAC-G0 and -G1), can be used to explore potential selection bias from any of the sources described above, and to inform sensitivity analyses to explore the extent of bias that might occur for any given analyses* *^20^* *. In this section we describe some of these approaches in general terms, as it is important to note that which methods are most appropriate to use will depend on the specific research questions being addressed and the likely source of selection (missing data). ^21,22^ Furthermore, ALSPAC data are available for scientists across the world to use. Whilst we highlight potential sources of selection bias in this cohort profile and will do so through conference presentations and the study website, we cannot dictate how other scientists approach these issues when using the study data. We will encourage others to explore issues of selection bias when using ALSPAC-G2 data and make details of their methods open access (including via linked Wellcome Open Research publications).*

*In our work we have found directed acyclic graphs ^21^ useful to illustrate specific sources of selection bias and their assumptions of any methods used to explore and control for these (see for example references ^20-24^). Selection bias results from missing data (for example, missing data due to not being recruited, being lost to follow-up or missing data on factors influencing different fertility practices across generations) thus methods for dealing with missing data are appropriate approaches to exploring and controlling for selection bias. Publication reporting guidelines, such as Strengthening The Reporting of Observational studies in Epidemiology (STROBE;* *https://strobe-statement.org/index.php?id=strobe-home* *) require authors to describe the extent of missing data and how potential bias from that has been explored and we strongly encourage users of ALSPAC to adhere to those reporting guidelines. Such reporting guidelines do not recommend specific approaches, e.g. for dealing with missing data, just that there is clarity about the issue. A common approach to dealing with missing data is complete case analyses (i.e. in which only participants with complete data on exposure, outcome and all covariables included in the main analyses are included in analyses). This is in general expected to be unbiased if missingness is not related to the outcome in the main analysis model conditioned on exposure and all covariables included in the main analyses. ^23^ Other commonly used methods for exploring and mitigating against selection bias include multiple imputation and inverse probability weighting, both of which assume that data are missing at random (MAR, missingness depends on observed data only ^23^). When analysing repeat measurements, e.g. trajectories of change in BMI or cognitive function, all participants with at least one value (of the variable that has been assessed repeatedly) can be included in the main analyses under a MAR assumption. Ideally, one would want to use more than one method, with differing assumptions, for exploring selection bias (bias due to missing data). In the preliminary cross-generational analyses presented here we compared results from complete case analyses to those using multiple imputation (including using two different approaches to the latter) and found similar results across all three approaches which increases confidence in these not being biased.*

*In general, research data that have been specifically collected as part of a study and linked data have complementary characteristics for exploring selection bias under the MAR assumption. Specific research data might predict missing values more strongly because it will have been collected at a similar time to variables with missing data (e.g. in sweeps of data collection) and will include some repeat collections of data obtained using the same methods across time. Such data area also likely to be obtained more extensively (e.g. several domains of socioeconomic position) and more precisely (e.g. DEXA scan total and body region fat and lean mass). However, specific research data may have more missingness due to loss to follow-up or some measures not being completed by some participants. Linked data is often available on all (or a large proportion) of participants.* *All generations of ALSPAC have both repeatedly assessed research and record linked data on many variables (see ^1,2^ and Figure 3), which considerably enhance the possibility for exploring and controlling for potential selection bias. Currently, ALSPAC-G0 and ALSPAC-G1 have been linked to obstetric-, primary-, secondary- and community health data, mortality and migration records and education data. Recent geocoding has also provided linkage to detailed environmental and area socioeconomic data. ^25^ Similar linkage for ALSPAC-G2 and the new (to ALSPAC) partners of existing ALSPAC-G1 is on-going and will be regularly updated. These data can be informative for methods assuming data are MAR (e.g. ^22^) and also where selection (missing information) is assumed to be missing not at random (i.e. where bias can remain even after taking into account observed predictors of missingness). ^24^ For example, using linked education data as proxy for IQ in ALSPAC-G1 it has been shown that methods assuming data were missing at random underestimated the strength of the association in comparison to the method assuming missing not at random. ^24^ In addition to linked data, genome-wide genetic data, which is available on a high proportion of ALSPAC-G0 and ALSPAC-G1 participants and will be on ALSPAC-G2 and the newly recruited partners of ALSPAC-G1, can be useful in exploring the nature of missing data. ^26^*

*Whilst we, ^22-24^ and others,(e.g. ^27^) have used the methods described above extensively in ALSPAC-G0 and ALSPAC-G1, their use for cross-generational analyses, in particular where the inclusion of siblings varies considerably across generations, has not yet been extensively explored. As demonstrated with ALSPAC-G2, such cross-generational analyses by necessity either restrict analyses to a narrow age range of parents in one generation (e.g. if we were to only continue to recruit ALSPAC-G2 for the next 5-years) or have participants recruited over a long period of years in one generation (as we hope to do with ALSPAC-G2).* *In the cross-generational analyses presented here we have been careful to describe the results as preliminary and illustrative. We undertook complete case analyses and used multiple imputation to explore potential selection due to missing data in the cross-generational comparisons presented here (Table 3). We used two approaches to multiple imputation, one including both generations in the same imputation analyses and one that separated the G0/G1 from the G1/G2 generations for the initial imputation. We found results were very similar across all three approaches. However, for simplicity, in these illustrative analyses we restricted the maternal age range in both generations to that of the ALSPAC-G1/G2 participants and to just one pregnancy per mother in each generation. This resulted in more of the G1/G2 pregnancies being excluded from analyses than G0/G1 pregnancies, which could have introduced bias that is not addressed in our multiple imputation analyses. In future research we plan to develop and use methods that can more fully explore the sources of selection bias in cross-generational analyses. This includes methods that combine multiple imputation and other missing data methods with  methods that appropriately take account of the complex multi-level structure of the data (i.e. repeat pregnancies within women across generations and potentially including complex family structures of siblings, cousins, nieces, nephews and so on). These methods and related analysis code will be disseminated widely via the ALSPAC website and via links to this cohort profile. We hope that other researchers will also use the unique multi-generational data of ALSPAC for exploring the likely impact of selection bias on this and other human multigeneration cohorts.* *” *( **Discussion section Page 24-27**)

Apologies, we were not sure just what the reviewer meant by “While creating a comparison cohort would be a useful adjunct to the recruitment of the G2 cohort this seems an unlikely reality in the short term given the bias in fertility rates and parental age at birth (as above) - if this is feasible then further details are required.”

We are not planning a comparator cohort for ALSPAC G2, but rather for three clinical cohorts that we are involved with (UK National Cleft Lip and Palate cohort, the Bristol IVF clinical cohort and a newly funded congenital heart disease cohort) ALSPAC-G2 will be used as a ‘healthy’ comparator group for some analyses in those clinical cohorts. For the follow-up of participants from the IVF and congenital heart disease cohorts are using/will use the ALSAPC clinic and ALSPAC-G2 protocols. Participants in these two cohorts are/will be initially be recruited from hospital and primary or secondary case antenatal clinics and have baseline data collected at those clinics and from record linkage. Follow-up will be through attendance at our research clinics where we also see ALSPAC participants, as well was with extensive record linkage.

 

- On the positive side making the multigenerational data resource available is excellent and the Data Access process is clearly articulated. Over the next two or more decades this will create a unique three generation resource - the challenge to users will be how to utilise appropriate methodologies to deal with the complexities of and delays in data capture over time.

**Response **We agree with this comment and hope that this profile highlights sufficiently many of the complexities. As we note in the revised version we have a programme of work on developing methods that that can address potential missing data and take advantage of the underlying complex family structure but also hope that other researchers will use the resource for methodological research as well as applied research and share these methods and code so that we can disseminate them via the study website and also link them to this profile.

# Peer review: Reviewer response for version 1

**DOI:** 10.21956/wellcomeopenres.16460.r35257

**Published:** 2019-04-18

**Authors:** Anna Vignoles

**Affiliations:** 1 Department of Education, University of Cambridge, Cambridge, UK

This is a very informative description of a particular cohort of children called ALSPAC-G2. These children are the grandchildren of the original ALSPAC-G0 cohort of mothers who were recruited into the ALSPAC study if they were due to give birth in the Avon area in 1991/1992. The children of the ALSPAC-G0 cohort (ALSPAC-G1, known to the public as Children of the 90s) have been followed into early adulthood and are now having children themselves. Their children (ALSPAC-G2 or Children of the Children of the 90s) are in the process of being recruited into the study, providing an intergenerational study spanning three generations. This paper describes ALSPAC-G2, the motivation for collecting data on these children and the key hypotheses that can be addressed with the data. It provides a vitally important resource guide for those considering using the data.

 The paper also reports some very interesting work that has already been done with the data and published elsewhere. This nicely illustrates the strengths of ALSPAC-G0/1/2 and certainly enhances the paper. However, it does mean that some of the findings reported cannot be verified with the data provided in the paper (though full references are given). This is why I have indicated that the detailed account of methods of analysis is only partly available in the paper. I do not see this as a problem however, and it is common in this type of paper to summarise others’ work in this way.

 The study is scientifically sound without further changes. However, I would note the following issues which the authors may wish to consider or comment on.

**Significant issues**

 The summary of the strengths and weaknesses of the data is very clear and comprehensive. In particular, the authors acknowledge the increasing selection bias that is likely to arise as ALSPAC recruits subsequent generations. It also discusses some of the potential risks associated with that. In particular, the data from ALSPAC-G2 will not be as representative of the Avon area as the original cohort. That said, the linkage with administrative data offers a potential opportunity not just to draw in additional phenotype data etc. but also to provide a source of external data that can be used to assess the extent of the representativeness of the ALSPAC data. This might be noted.

 As discussed, ALSPAC is a local cohort study but of course participants move away. Participants are offered travel costs to the Bristol based clinics for the assessments so technically even if a cohort member of ALSPAC-G1 has moved away, they can participate. However, it would be useful to note the proportion of ALSPAC-G1 parents who are no longer living in Bristol and response rates to ALSPAC-G2 across those who have and have not moved away. This would help quantify any potential selection bias on this particular issue.

 The comparisons of young mothers across the two generations do not discuss the fact that selection into early motherhood may be driven by different (unobserved) factors across the two generations. If, for example, attitudes have changed and early childbirth is now viewed more negatively by women in the more recent generation (even when comparing women with similar characteristics), this may lead to selection bias into young parenthood. This may be particularly relevant for some outcomes, such as maternal postnatal depression. There is no way to account for this empirically but it might be noted.

 Table 3. The comparison of age at first pregnancy between the two cohorts (ALSPAC-G0/G1, and ALSPAC-G1/G2) depends crucially on restricting the sample to age 19-26. Otherwise the mean age at first birth statistic will be misleading because, given that ALSPAC-G1 is a cohort of roughly the same age, we will not know the true mean age of mother at birth of first child until some more years have passed and others in the ALSPAC-G1 generation have had their children. This is carefully footnoted but I wonder whether the age restriction might usefully appear in the title of the table.

**Minor issues**

 The abstract mentions data collection and notes that ALSPAC-G2 collects a wide range of “social” data. In the main body of the text this is sometimes changed to “socioeconomic”. The latter is more accurate and might replace the word “social” throughout.

 Bullet 4 on page 3 is somewhat strangely worded. The intention of ALSPAC-G2 is to undertake intergenerational analyses of the factors influencing decisions by ALSPAC-G0 compared to ALSPAC-G1 about whether, and when, to start a family, parenting patterns, child development, parental and child mental and physical health and ‘work-family/outside of work’ balance. This main aim might come earlier in the bullet before discussing the variables that are in the data?

 There is a typographical error on page 3 under the section “Who is in the cohort?”. “We aim is to recruit..”

 References to the Bristol IVF and congenital heart disease cohorts might be provided to help the reader.

I confirm that I have read this submission and believe that I have an appropriate level of expertise to confirm that it is of an acceptable scientific standard.

# Response

**Published:** 2019-11-14

**Authors:** Deborah Lawlor

**Affiliations:** University of Bristol, UK

This is a very informative description of a particular cohort of children called ALSPAC-G2. These children are the grandchildren of the original ALSPAC-G0 cohort of mothers who were recruited into the ALSPAC study if they were due to give birth in the Avon area in 1991/1992. The children of the ALSPAC-G0 cohort (ALSPAC-G1, known to the public as Children of the 90s) have been followed into early adulthood and are now having children themselves. Their children (ALSPAC-G2 or Children of the Children of the 90s) are in the process of being recruited into the study, providing an intergenerational study spanning three generations. This paper describes ALSPAC-G2, the motivation for collecting data on these children and generations. This paper describes ALSPAC-G2, the motivation for collecting data on these children and the key hypotheses that can be addressed with the data. It provides a vitally important resource guide for those considering using the data.

The paper also reports some very interesting work that has already been done with the data and

published elsewhere. This nicely illustrates the strengths of ALSPAC-G0/1/2 and certainly enhances the paper. However, it does mean that some of the findings reported cannot be verified with the data provided in the paper (though full references are given). This is why I have indicated that the detailed account of methods of analysis is only partly available in the paper. I do not see this as a problem however, and it is common in this type of paper to summarise others’ work in this way.

The study is scientifically sound without further changes. However, I would note the following issues

which the authors may wish to consider or comment on.

**Significant issues**

- The summary of the strengths and weaknesses of the data is very clear and comprehensive. In particular, the authors acknowledge the increasing selection bias that is likely to arise as ALSPAC recruits subsequent generations. It also discusses some of the potential risks associated with that. In particular, the data from ALSPAC-G2 will not be as representative of the Avon area as the original cohort. That said, the linkage with administrative data offers a potential opportunity not just to draw in additional phenotype data etc. but also to provide a source of external data that can be used to assess the extent of the representativeness of the ALSPAC data. This might be noted.

**Response**

We thank the reviewer for these comments. In response to reviewer 1, in the revised paper we have extended the section on strengths and limitations and use subheadings covering three key areas – selection bias, representativeness, and measurements across generations. Below we have copied the now extended discussion of the first two of these, which we hope provide sufficient information to inform future users of the data. Within this we have also tried to encourage future users to also share methodological insights into selection bias/representativeness within this resource. These will also have potential value for other studies with complex family data.

***“Selection bias***

*There are a number of sources of potential selection bias in ALSPAC-G2* *^20^* *. Some of these, such as incomplete recruitment and loss to follow-up, are common to most prospective cohort studies, whereas others, such as the current recruitment at a relatively young age and the focus of analyses in cross-generational effects, are more specific to ALSPAC-G2. Below we describe some of the key potential sources of selection bias, general methods that might be used to mitigate against some of the selection mechanisms, and data and analytical approaches that could be used to explore and control for potential selection bias. It is important to note that appropriate methods for exploring and controlling for selection bias are specific to the analyses being undertaken. Furthermore, ALSPAC is a resource for the global scientific community to use and we do not dictate how scientists using this resource complete their analyses. Whilst we make suggestions here that might be used to explore selection bias, researchers must decide how they want to approach this for the specific questions they are exploring with these data.*

*Our response is 56% of those invited (53% overall) and respondents are more likely to be those already engaged with ALSPAC as indicated by being more likely to have participated in the two most recent clinic assessments for all ALSPAC-G1 participants. Once recruited a high proportion remain actively engaged with follow-up, though the extent to which this will continue as the cohort ages is unknown. Currently, ALSPAC-G2 are children being born to relatively young parents (mean 22 years (range 17-26)); though the age range of parents will increase with continued follow-up. Whilst cross-generational comparisons that have already been published and/or presented in this paper are age matched, it is possible that the factors that influence starting a family at a relatively young age differ across generations. If these factors are unknown or unmeasured (and therefore cannot be controlled for) there may be bias in the cross generational comparisons. This may be further complicated by secular trends in some of these factors. For example, preliminary analyses presented here suggest that age matched ALSPAC-G1 mothers with an ALSPAC-G2 child are more educated than were their ALSPAC-G0 mother’s generation when recruited during their pregnancy ~30years ago. This difference may be due to true differences between UK pregnant women across generations. For example, there may have been a reduction in the stigma attached to becoming pregnant at a young age and hence a reduction in the association of education/socioeconomic position with it. ALSPAC-G1 mothers may also be truly more educated than their parent’s generation as a result of changes in education policy and secular trends related to that policy. Over the last 20-30 years there has been a doubling of the proportion of people obtaining a University degree in the UK, stimulated by policy changes, including previous further education establishments becoming Universities in the 1990s. Alternatively these cross-generational differences in education could be due to selection bias. This is supported by our finding that those ALSPAC-G1 parents recruited as pregnant women or parents to ALSPAC-G2 are more engaged with the study than those not recruited (Table 2), and that ALSPAC-G1 participants who are more engaged with the study are on average more educated than those less engaged ^1,2^.*

*At the time we began recruitment, ALSPAC-G1 participants were aged 19–21 years and some had already become parents. Thus, whilst the cohort is currently of relatively young parents, we do not have detailed pregnancy data on those who became pregnant before 19-years. To maximise participation of all ALSPAC-G2 we will continue to recruit participants at different ages. Forthose ALSPAC-G2 participants recruited after birth, either because they were born prior to when we began recruitment or because their parents only decided to join ALSPAC-G2 when their child/children were older, their mother’s pregnancy information and their birth/infancy information will be limited to what we can obtain from record linkage. With the extensive linkage that is now possible, including primary-, secondary- and community health care, education and environmental linkage we will still have a lot of information on these pregnancies and the resulting infants. Furthermore, the proportion recruited in pregnancy has increased over time and once a family have joined ALSPAC-G2, subsequent children are mostly recruited during their mother’s pregnancy.*

*To mitigate against some of these sources of bias we aim to minimise participant burden by using record linkage and remote data collection as much as possible. The ability to do this is supported by the existence of a National Health Service in the UK and large-scale initiatives across UK research funders for supporting ethical and appropriate access to anonymised health, environmental, and socioeconomic data. Linked data can also be used for describing the likely nature of any selection bias and informing sensitivity analyses.*

*Both existing research and record linkage data, including on grandparents and parents (ALSPAC-G0 and -G1), can be used to explore potential selection bias from any of the sources described above, and to inform sensitivity analyses to explore the extent of bias that might occur for any given analyses* *^20^* *. In this section we describe some of these approaches in general terms, as it is important to note that which methods are most appropriate to use will depend on the specific research questions being addressed and the likely source of selection (missing data). ^21,22^ Furthermore, ALSPAC data are available for scientists across the world to use. Whilst we highlight potential sources of selection bias in this cohort profile and will do so through conference presentations and the study website, we cannot dictate how other scientists approach these issues when using the study data. We will encourage others to explore issues of selection bias when using ALSPAC-G2 data and make details of their methods open access (including via linked Wellcome Open Research publications).*

*In our work we have found directed acyclic graphs ^21^ useful to illustrate specific sources of selection bias and their assumptions of any methods used to explore and control for these (see for example references ^20-24^). Selection bias results from missing data (for example, missing data due to not being recruited, being lost to follow-up or missing data on factors influencing different fertility practices across generations) thus methods for dealing with missing data are appropriate approaches to exploring and controlling for selection bias. Publication reporting guidelines, such as Strengthening The Reporting of Observational studies in Epidemiology (STROBE;* *https://strobe-statement.org/index.php?id=strobe-home* *) require authors to describe the extent of missing data and how potential bias from that has been explored and we strongly encourage users of ALSPAC to adhere to those reporting guidelines. Such reporting guidelines do not recommend specific approaches, e.g. for dealing with missing data, just that there is clarity about the issue. A common approach to dealing with missing data is complete case analyses (i.e. in which only participants with complete data on exposure, outcome and all covariables included in the main analyses are included in analyses). This is in general expected to be unbiased if missingness is not related to the outcome in the main analysis model conditioned on exposure and all covariables included in the main analyses. ^23^ Other commonly used methods for exploring and mitigating against selection bias include multiple imputation and inverse probability weighting, both of which assume that data are missing at random (MAR, missingness depends on observed data only ^23^). When analysing repeat measurements, e.g. trajectories of change in BMI or cognitive function, all participants with at least one value (of the variable that has been assessed repeatedly) can be included in the main analyses under a MAR assumption. Ideally, one would want to use more than one method, with differing assumptions, for exploring selection bias (bias due to missing data). In the preliminary cross-generational analyses presented here we compared results from complete case analyses to those using multiple imputation (including using two different approaches to the latter) and found similar results across all three approaches which increases confidence in these not being biased.*

*In general, research data that have been specifically collected as part of a study and linked data have complementary characteristics for exploring selection bias under the MAR assumption. Specific research data might predict missing values more strongly because it will have been collected at a similar time to variables with missing data (e.g. in sweeps of data collection) and will include some repeat collections of data obtained using the same methods across time. Such data area also likely to be obtained more extensively (e.g. several domains of socioeconomic position) and more precisely (e.g. DEXA scan total and body region fat and lean mass). However, specific research data may have more missingness due to loss to follow-up or some measures not being completed by some participants. Linked data is often available on all (or a large proportion) of participants.* *All generations of ALSPAC have both repeatedly assessed research and record linked data on many variables (see ^1,2^ and Figure 3), which considerably enhance the possibility for exploring and controlling for potential selection bias. Currently, ALSPAC-G0 and ALSPAC-G1 have been linked to obstetric-, primary-, secondary- and community health data, mortality and migration records and education data. Recent geocoding has also provided linkage to detailed environmental and area socioeconomic data. ^25^ Similar linkage for ALSPAC-G2 and the new (to ALSPAC) partners of existing ALSPAC-G1 is on-going and will be regularly updated. These data can be informative for methods assuming data are MAR (e.g. ^22^) and also where selection (missing information) is assumed to be missing not at random (i.e. where bias can remain even after taking into account observed predictors of missingness). ^24^ For example, using linked education data as proxy for IQ in ALSPAC-G1 it has been shown that methods assuming data were missing at random underestimated the strength of the association in comparison to the method assuming missing not at random. ^24^ In addition to linked data, genome-wide genetic data, which is available on a high proportion of ALSPAC-G0 and ALSPAC-G1 participants and will be on ALSPAC-G2 and the newly recruited partners of ALSPAC-G1, can be useful in exploring the nature of missing data. ^26^*

*Whilst we, ^22-24^ and others,(e.g. ^27^) have used the methods described above extensively in ALSPAC-G0 and ALSPAC-G1, their use for cross-generational analyses, in particular where the inclusion of siblings varies considerably across generations, has not yet been extensively explored. As demonstrated with ALSPAC-G2, such cross-generational analyses by necessity either restrict analyses to a narrow age range of parents in one generation (e.g. if we were to only continue to recruit ALSPAC-G2 for the next 5-years) or have participants recruited over a long period of years in one generation (as we hope to do with ALSPAC-G2).* *In the cross-generational analyses presented here we have been careful to describe the results as preliminary and illustrative. We undertook complete case analyses and used multiple imputation to explore potential selection due to missing data in the cross-generational comparisons presented here (Table 3). We used two approaches to multiple imputation, one including both generations in the same imputation analyses and one that separated the G0/G1 from the G1/G2 generations for the initial imputation. We found results were very similar across all three approaches. However, for simplicity, in these illustrative analyses we restricted the maternal age range in both generations to that of the ALSPAC-G1/G2 participants and to just one pregnancy per mother in each generation. This resulted in more of the G1/G2 pregnancies being excluded from analyses than G0/G1 pregnancies, which could have introduced bias that is not addressed in our multiple imputation analyses. In future research we plan to develop and use methods that can more fully explore the sources of selection bias in cross-generational analyses. This includes methods that combine multiple imputation and other missing data methods with  methods that appropriately take account of the complex multi-level structure of the data (i.e. repeat pregnancies within women across generations and potentially including complex family structures of siblings, cousins, nieces, nephews and so on). These methods and related analysis code will be disseminated widely via the ALSPAC website and via links to this cohort profile. We hope that other researchers will also use the unique multi-generational data of ALSPAC for exploring the likely impact of selection bias on this and other human multigeneration cohorts.*

***Representativeness of different generations to the target population***

*The original women recruited to ALSPAC (i.e. ALSPAC-G0) had similar socioeconomic characteristics and ethnic backgrounds to those of the population of the former county of Avon (South West region of England). ^2^ They were more affluent and more likely to be White European than the whole of the UK in the early 1990s, which reflects the difference between the population in the South West of England where the ALSAPC resource is based and the rest of the UK. ^2^ At age 16-years their children (ALSPAC-G1 – i.e. the parents of ALSPAC-G2) had somewhat higher school test scores than the national average, with this difference increasing in sub-samples based on increasing loss to follow-up. ^1 ^As recruitment of ALSPAC-G2 has been higher for those whose ALSPAC-G1 parents were most engaged with the study it is possible that over time ALSPAC-G2 will show greater differences to national averages. We will monitor that through the extensive record linkage, including data on education. Whilst selection bias and representativeness are sometimes treated as if they reflect the same concept, here we consider that lack of representativeness to the target population may bias estimates of prevalence but does not necessarily influence association or causal effect estimates. There have been considerable debates on this topic (see for example ^28-33^), with evidence that representativeness is not necessary to obtain unbiased estimates of association or effect, ^28-32^ though this will depend on the extent of selection into a study and the availability of data to predict this. ^20,33^ In this regard a key strength of ALSPAC-G2 is the existence of extensive data on previous generations and linkage to national data across all generations. ^33^*

*With respect to analyses that might only be done within the ALSPAC-G2 cohort currently, we would consider association or causal effect estimates to generalise to couples becoming pregnant at a relatively young age. This would be consistent with other pregnancy studies that recruit at younger ages and/or only recruit nulliparous women (such as ^34,35^). Once we have had the opportunity to invite all of ALSPAC-G2 we will have a unique study in which to explore the relationships of age at which couples become pregnant/parents across a wide age range within a relatively homogeneous cohort in terms of date and place of birth.” (* **Discussion section of the paper page 24-28 of clean version** *)*

- As discussed, ALSPAC is a local cohort study but of course participants move away. Participants are offered travel costs to the Bristol based clinics for the assessments so technically even if a cohort member of ALSPAC-G1 has moved away, they can participate. However, it would be useful to note the proportion of ALSPAC-G1 parents who are no longer living in Bristol and response rates to ALSPAC-G2 across those who have and have not moved away. This would help quantify any potential selection bias on this particular issue.

**Response**

We agree this is a really useful suggestion and have now added to Table 2 in the revision a row that shows the proportion of people living in the Bristol area (BS postcode) at the time that they were invited to join the study by whether they participated or did not. There is no difference between the two, with 82% of the respondents and 83% of the non-respondents being resident in Bristol. Before doing these additional analyses (i.e. comparing Bristol residency between respondents and non-respondents) we *a priori* agreed that this was valuable analyses to undertake and that residence in Bristol at the time of recruitment could be a predictor of missingness and therefore *a priori* we would include it in the prediction models for the multiple imputation analyses that are presented in both Table 2 and Table 3. As expected given the similarity between respondents and non-respondents with respect to living in Bristol none of the multiple imputation results changed in any substantial way but there are small changes that have been made to the results in these tables in the revised version.

- The comparisons of young mothers across the two generations do not discuss the fact that selection into early motherhood may be driven by different (unobserved) factors across the two generations. If, for example, attitudes have changed and early childbirth is now viewed more negatively by women in the more recent generation (even when comparing women with similar characteristics), this may lead to selection bias into young parenthood. This may be particularly relevant for some outcomes, such as maternal postnatal depression. There is no way to account for this empirically but it might be noted.

**Response**

We agree, and in the extended section on selection bias (see above) this is now noted more clearly.

- Table 3. The comparison of age at first pregnancy between the two cohorts (ALSPAC-G0/G1, and ALSPAC-G1/G2) depends crucially on restricting the sample to age 19-26. Otherwise the mean age at first birth statistic will be misleading because, given that ALSPAC-G1 is a cohort of roughly the same age, we will not know the true mean age of mother at birth of first child until some more years have passed and others in the ALSPAC-G1 generation have had their children. This is carefully footnoted but I wonder whether the age restriction might usefully appear in the title of the table.

**Response**

Thank you, we have now made this change to the title of Table 3

**Minor issues**

- The abstract mentions data collection and notes that ALSPAC-G2 collects a wide range of “social” data. In the main body of the text this is sometimes changed to “socioeconomic”. The latter is more accurate and might replace the word “social” throughout.

**Response**

We have changed most of the times we use ‘social’ to socioeconomic; in particular we have done this when we are referring to socioeconomic data, such as occupational social class or education. We still use social for e.g. ‘social life’ and ‘social media’.

- Bullet 4 on page 3 is somewhat strangely worded. The intention of ALSPAC-G2 is to undertakeintergenerational analyses of the factors influencing decisions by ALSPAC-G0 compared to ALSPAC-G1 about whether, and when, to start a family, parenting patterns, child development, parental and child mental and physical health and ‘work-family/outside of work’ balance. This main aim might come earlier in the bullet before discussing the variables that are in the data?

**Response**

We have made this change and reworded this section in the revised paper so that we hope it is now clearer. This now reads as follows:

*“3.* *Understanding the impact of major changes that have occurred over the last 20-25 years and how these have influenced differences between ALSPAC-G0 and ALSPAC-G1 in relation to whether, and when, to start a family, parenting patterns, and ‘work-family/outside of work’ balance and of these on differences in health and development of ALSPAC-G1 and ALSPAC-G2. These changes have been unprecedented and include changes in environmental (e.g. climate change, air-pollution), societal (e.g. different methods of communication, changing patterns of between and within country migration), political (e.g. distrust of experts, departures from traditional political parties, changes in gender politics, retirement age and types of employment) technological (e.g. widespread use of information technology and the emergence of artificial intelligence) and lifestyle (e.g. sedentary behaviour, reduced smoking and alcohol consumption, increased vaping) factors. For example, ALSPAC-G0 participants mostly finished their education and started their careers before the internet was invented. In its current recognisable form, widespread availability of the internet began in 1990 just before ALSPAC-G1 were born but its reach into primary schools will have been limited for this generation. In contrast, ALSPAC-G2 are likely to spend their whole lives extensively using the internet for their education, work and social life.”* **(introduction section page 3)**  

- There is a typographical error on page 3 under the section “Who is in the cohort?”. “We aim is to recruit..”

**Response**

This has now been corrected, thank you.

- References to the Bristol IVF and congenital heart disease cohorts might be provided to help the reader.

**Response**

We have now added these for the cleft lip and palate and the IVF cohort and note that currently there is no website for the congenital heart disease cohort.
